# Chromosome-scale and haplotype-resolved genome assembly of *Populus trichocarpa*

**DOI:** 10.1093/hr/uhaf012

**Published:** 2025-01-15

**Authors:** Wentao Gao, Sui Wang, Tao Jiang, Heng Hu, Runtian Gao, Murong Zhou, Guohua Wang

**Affiliations:** College of Life Sciences, Northeast Forestry University, Harbin, Heilongjiang 150040, China; National Key Laboratory of Smart Farm Technologies and Systems, Northeast Agricultural University, Harbin, Heilongjiang 150038, China; Key Laboratory of Soybean Biology of Chinese Education Ministry, Northeast Agricultural University, Harbin, Heilongjiang 150038, China; School of Computer Science and Technology, Harbin Institute of Technology, Harbin, Heilongjiang 150001, China; College of Life Sciences, Northeast Forestry University, Harbin, Heilongjiang 150040, China; College of Life Sciences, Northeast Forestry University, Harbin, Heilongjiang 150040, China; College of Life Sciences, Northeast Forestry University, Harbin, Heilongjiang 150040, China; College of Computer and Control Engineering, Northeast Forestry University, Harbin, Heilongjiang 150040, China; State Key Laboratory of Tree Genetics and Breeding, Northeast Forestry University, Harbin, Heilongjiang 150040, China

## Abstract

*Populus trichocarpa,* a pivotal model organism for woody transgenic research, not only garners substantial scientific interest but plays an integral role in forestry economics. Previous genomic assemblies of *P. trichocarpa* predominantly treated its heterozygous genome as homozygous, thereby neglecting crucial haplotypic diversity. Leveraging the high-fidelity (HiFi) sequencing capabilities of PacBio sequencing and the chromosome conformation capture insights provided by Illumina's Hi-C technique, this study is the first to achieve a near telomere-to-telomere assembly of both paternal and maternal haplotypes in *P. trichocarpa*. Comparative genomic analysis between these haplotypes has uncovered several allelic variants and pathways critical for trait determination through allele-specific expression. Furthermore, utilizing RNA-seq data from multiple tissues, this investigation has detailed the tissue-specific expression patterns of the leucine-rich repeat gene family, which are essential in mediating plant signal transduction and developmental regulation. Our results not only illuminate the functional genomics landscape of *P. trichocarpa* but also provide invaluable theoretical underpinnings for the genetic improvement of woody plants and a robust framework for exploring genetic variability and allelic expression disparities in arboreal species.

## Introduction

Forest ecosystems play a pivotal role in global carbon cycling and mitigating climate change. *Populus trichocarpa*, a poplar native to North America and belonging to the family Salicaceae, emerges as an archetypal model species for woody plant research. This species features a moderately sized genome and rapid growth traits, making it an exemplary candidate for studying wood formation, growth dynamics, and fundamental biological processes in trees. *P. trichocarpa* was the first tree species to have its genome sequenced in 2006 [1], and it serves as a vital raw material for bioenergy production.

Advances in technology have facilitated the genomic elucidation of *P. trichocarpa*, its related species, and hybrids. Investigations have systematically identified and analyzed orphan genes within *P. trichocarpa*, uncovering evidence of *de novo* gene evolution and elucidating potential functions of these genes in stress and defense responses. Additionally, by comparing collinear regions among *P. trichocarpa*, *P. deltoides*, and *Salix purpurea*, the evolutionary trajectory of orphan genes has been reconstructed, with expression quantitative trait loci (eQTL) mapping employed to delineate their regulatory networks [2]. Recent genomic updates for *P. euphratica* (v2.0) have revealed significant expansion of Gypsy elements, likely facilitating adaptation to extreme environments [3]. This species also exhibits extensive structural rearrangements, such as translocations and inversions, providing key insights into its environmental adaptability. In other related studies, an enhanced genome for European black poplar has been released, leveraging long-read sequencing, optical mapping, and genetic mapping techniques (Schiffthaler et al. 2019). Additionally, genomes for tropical Salicaceae species *P. qiongdaoensis* [4] and North Hemisphere's *P. deltoides* [5] have been sequenced.

Moreover, genomes of several hybrids and subspecies within the Populus genus have been published, including the triploid hybrid ‘Yinzhong poplar’ [6] and the haploid reference genome of *P.tomentosa Carr.* [7]. Heterozygous tree genomes for *P. tremula L.* and *Populus tremuloides Michx* were also released in 2018 [8]. Recent studies employing trio-binning design and HiFi sequencing technologies have produced the T2T haploid genome for '84 K' (*Populus alba* × *Populus tremula var. glandulosa*), revealing significant allelic expression differences [9]. The application of machine learning models to predict allele-specific expression (ASE) highlighted the role of CHG methylation, sequence variations, and transposable elements as key factors influencing expression differences. These findings offer new tools and perspectives for functional studies and hybrid trait analysis of poplar trees. In addition to these advancements, multiple studies on poplar and pan-genomics have been conducted. A recent study constructed a super-pangenome comprising 19 genomes of the Populus genus, revealing the pivotal role of private genes in local environmental and climatic adaptation. Integrative analysis of the pan-genome, transcriptome, methylome, and chromatin architecture demonstrated that the evolution of pan and duplicate genes is closely associated with their regulation and epigenetic structure [10]. Previously, genomic sequencing of 10 species across five classification groups within the Populus genus identified 71 million genomic variants, unveiling new correlations between single nucleotide polymorphisms (SNPs), structural variations (SVs), and insertions-deletions (InDels) with SVs [11]. These efforts have significantly enriched the genetic database for species within the Populus genus, providing precise reference resources for molecular experiments and breeding improvements in poplar trees.

With the rapid advancement of third-generation sequencing technologies, unprecedentedly detailed and accurate genomic data are now obtainable. In particular, high-fidelity (HiFi) sequencing technologies, notable for their extended read lengths and high precision, have emerged as powerful new tools for in-depth analysis of genomic structures. Moreover, recent advancements in genomic assembly algorithms have greatly facilitated the assembly of individual chromosomal copies, including both homologous and heterologous chromosomes, offering new avenues for research [12]. Specifically, a strategy known as Trio binning, which utilizes sequencing data from parents, provides a novel solution for haplotype-resolved assembly [13], enabling effective resolution of haploid genomes in diploid species. Additionally, research on tetraploid potatoes has demonstrated a gamete-binning assembly strategy dependent on gamete sequencing data [14], while another haplotype-resolving approach leverages single-cell sequencing data for precise localization in polyploid genomes [15].

**Figure 1:**
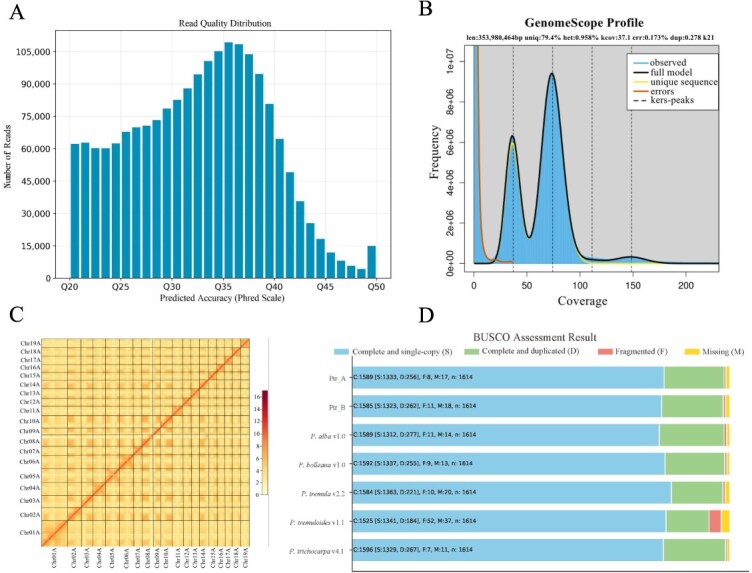
Genome assembly quality and completeness estimation. (A) HiFi reads quality distribution. (B) Result of genome survey. (C) Hi-C interaction matrix maps of Ptr_A. (D) BUSCO analysis of the genomes from *P. alba*, *P. bolleana*, *P. tremula*, *P. tremuloides*, and the heterozygous genome of *P. trichocarpa*

**Figure 2:**
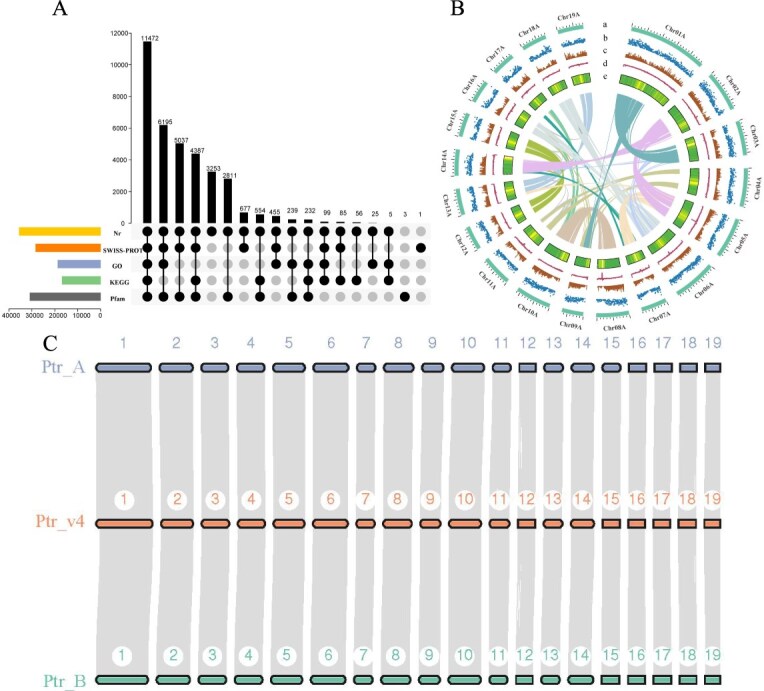
Haplotype-resolved near T2T *P. trichocarpa* genome assembly. (A) Gene function annotation of Ptr_A. (B) Chromosome features and distribution of nucleotide polymorphisms. In Circos plots from outmost to innermost: (a) chromosome size, (b) InDel density in Ptr_A, (c) SNP density, (d) GC content, and (e) gene density; lines in the center indicate syntenic gene pairs. (C) Comparison of chromosome structure between Ptr_A, Ptr_B haplotype genomes, and *P. trichocarpa* v4.1 (Ptr v4.1)

Despite extensive research involving *P. trichocarpa* and its close relatives, to date, there has been no report on the haploid genome resolution of this species. As a naturally hybrid species, the resolution of haploid reference genomes in *P. trichocarpa* not only reveals the genomic composition and structural variations of both parents but also elucidates how interspecific hybridization enhances genomic diversity. Through haplotype assembly, specific transcriptomic analyses can be conducted to identify genes with differential expression between the two parents [16, 17], and to study how expression differences in homologous genes across different haplotypes impact phenotypes. Overall, haplotype assembly provides a potent new tool for analyzing the genetic diversity and complexity of tree hybrid species, and is expected to significantly advance the fields of molecular genetics and breeding in forestry.

This study presents the first report of a chromosome-scale of the *P. trichocarpa* haploid genome. Further analyses in this study include the distribution of transposable elements (TEs) within the haploid *P. trichocarpa* genome, as well as allelic expression differences, particularly the differential expression patterns of the leucine-rich repeat (LRR) gene family across different tissues. This work not only showcases the value of high-quality *P. trichocarpa* genome assembly but also highlights its potential applications in the genetic breeding of forest plants.

## Results

### Genome sequencing and assembly

Sequencing of nuclear DNA from *P. trichocarpa* tissue culture seedlings was carried out using the PacBio Sequel II sequencing platform, resulting in 59.98 Gb of HiFi long-read sequencing data with an average read length of 16.5 Kb and a sequencing depth of approximately 78× (Fig. 1A and Supplemental Table S1). In addition, 250.8 Gb of Hi-C sequencing data was generated using the PE150 sequencing method (Supplemental Fig. S1 and Supplemental Table S2). Initially, a genome survey of *P. trichocarpa* PacBio HiFi data was performed using the *k*-mer analysis tool GenomeScope (Supplemental Fig. S2) [18]. The genome assessment results indicated that the genome size of *P. trichocarpa* was approximately 378.6 Mb, with a heterozygosity of 0.97% (Fig. 1B). Preliminary assemblies of the two *P. trichocarpa* haplotypes were generated using PacBio HiFi data and the Hi-C mode of the Hifiasm assembly tool [19, 20]. The Hi-C data were employed to anchor the preliminary haplotype assemblies to the chromosome level (Fig. 1C and Supplemental Figs S9 and S10), using the YaHS tool [20] in this process (Fig. 2B). Following gap filling, two haplotype genomes (2*n* = 38) were obtained, with sizes of 391.76 and 397.43 Mb, respectively. The two haplotype genomes were designated Ptr_A (Chr##A) and Ptr_B (Chr##B).

### Gaps filled and identification of telomeres and centromeres

In this study, we utilized PacBio HiFi sequencing data aligned to a haplotype-resolved assembly to address the gap regions within the Ptr_A and Ptr_B genomes. The Ptr_A genome has been largely resolved, with six gaps remaining, while the Ptr_B genome is nearly complete, with two gaps located on chromosomes 15B and 17B. All 18 chromosomes of Ptr_A were successfully assembled, although chromosome 14A is missing one telomere, highlighting a specific challenge in telomere assembly (Supplemental Figs S5-S8).

The assembly's high quality was affirmed by the successful identification of telomeres and centromeres, demonstrating near telomere-to-telomere (T2T) completeness. The use of the telomere repeat monomer AAACCCT facilitated the detection of complete telomeres on 17 chromosomes in Ptr_A, with partial assemblies noted in chromosomes 08A and 14A.

Furthermore, our analysis of sequence repetition within the tandem repeat and complex centromeric regions identified 19 centromeres in each haplotype. The centromeric regions in Ptr_A range from 338 987 to 2 290 206 bp, with an average of 1 169 967 bp (Table 1 and Supplemental Table S3). This comprehensive genomic assembly highlights the effectiveness of long-read sequencing technologies in resolving complex genomic structures.

### Evaluation of genome assembly

Genome integrity was rigorously evaluated using the embryophyta single-copy gene set, with the Ptr_A and Ptr_B haplotypes exhibiting coverage of 98.5% and 98.2% of these genes, respectively (Supplemental Tables S4 and S5). Quantitative assessment of the haplotype assemblies was conducted using the QUAST software [21], resulting in N50 metrics of 21.8 Mb for Ptr_A and 20.9 Mb for Ptr_B. These metrics are detailed in Table 2. Additionally, the correlation between the two haplotype genomes also provides evidence supporting the accuracy of our assembly and annotation (Supplemental Figs S13–S16).

**Table 1:** Centromeres in Ptr_A genome.

**Chromosome**	**Start**	**End**	**Length (bp)**
Chr01A	17 067 855	18 388 043	1 320 189
Chr02A	18 390 105	18 729 091	338 987
Chr03A	5 217 836	6 185 951	968 116
Chr04A	12 286 369	12 851 388	565 020
Chr05A	14 189 618	15 967 465	1 777 848
Chr06A	14 736 384	15 244 919	508 536
Chr07A	6 684 151	8 355 330	1 671 180
Chr08A	14 922 861	17 213 066	2 290 206
Chr09A	269 249	743 673	474 424
Chr10A	4 426 905	5 069 397	642 493
Chr11A	8 573 322	10 862 178	2 288 857
Chr12A	7 271 559	8 008 585	737 027
Chr13A	8 111 037	10 058 058	1 947 022
Chr14A	14 179 935	16 551 967	2 372 033
Chr15A	5 233 979	6 002 367	768 389
Chr16A	8 080 279	8 498 125	417 847
Chr17A	5 500 200	5 737 440	237 241
Chr18A	6 047 905	7 375 199	1 327 295
Chr19A	7 022 119	7 979 474	957 356

**Table 2:** *P. trichocarpa* genome assembly statistics.

**Parameter**	**Ptr_A**	**Ptr_B**
Genome size (Mb)	391.76	397.43
Number of scaffolds	19	19
N50 (Mb)	21.8	20.9
L50	7	8
N75 (Mb)	16.3	16.6
L75	13	13
Number of telomeres	37	34
Number of centromeres	19	19
GC count (%)	33.86	33.91
BUSCO (%)	98.5	98.2

### Genome annotation

In the domains of transposable elements (TEs) and repeat annotation, the quality and contiguity of repeat sequence assemblies were evaluated using the long terminal repeat (LTR) assembly Index (LAI) [22]. The LAI values for the Ptr_A and Ptr_B assemblies were 18.48 and 18.72, respectively, indicating reference-level quality. These results suggest that the haploid assemblies demonstrated continuity in the repetitive fractions of the genome, comparable to that of the reference sequences. Additionally, the EDTA pipeline [22] was employed for comprehensive annotation of repeat sequences, including transposons. In the Ptr_A genome, repeats and TEs constituted approximately 36.02% of the total genome size, with LTR retrotransposons accounting for about 16.07%. Terminal inverted repeats and Helitron DNA transposons represented 17.02% of the genome. Minor variations were observed in the repetitive regions across the two haploid genomes. Detailed annotation results are provided in Table 3.

**Table 3:** Classification of repetitive elements in the Ptr_A and Ptr_B assembly and Ptr v4.1.

**Category**	**Ptr_A**	**Ptr_B**	**Ptr v4.1**
LTR/Copia	19 485 (3.64%)	16 532 (3.43%)	17 798 (3.62%)
LTR/Gypsy	46 218 (9.00%)	42 329 (9.14%)	41 831 (8.02%)
LTR/Other	38 168 (3.73%)	39 236 (3.51%)	32 006 (3.41%)
TIR/CACTA	10 753 (0.86%)	14 085 (1.27%)	20 860 (2.00%)
TIR/Mutator	37 396 (2.26%)	23 155 (3.08%)	14 368 (1.26%)
TIR/PIF_Harbinger	9538 (0.58%)	5810 (0.36%)	7215 (0.38%)
TIR/Tc1_Mariner	2129 (0.17%)	1506 (0.13%)	3862 (0.32%)
TIR/hAT	13 312 (1.53%)	14 172 (1.08%)	14 976 (1.24%)
DNA/helitron	114 412 (11.62%)	123 604 (12.63%)	117 043 (11.22%)
Total	328 853 (36.02%)	320 326 (37.30%)	318 979 (34.54%)

The contents of the cells were counts (percentage of sequence %), LTR, long terminal repeat; TE, transposable element; TIR, terminal inverted repeat.

Gene annotation was performed by integrating *de novo*, homology-based, and transcript-based predictions to establish the genetic architecture of *P. trichocarpa,* primarily utilizing the braker3 pipeline [23]. Within the haploid genome Ptr_A, 30 089 genes, 35 979 mRNAs, 207 045 exons, and 171 066 introns were annotated, with the average lengths of the mRNA, exons, and introns being 3375.7, 234.2, and 426.5 bp, respectively. Detailed annotation information is shown in Table 4. The haploid genome Ptr_B was annotated with 30 331 genes, 36 192 mRNAs, 207 726 exons and 171 534 introns, with average mRNA, exon and intron lengths of 3362.5, 234.1 and 426 bp, respectively. These findings indicate a fundamental similarity in gene frequency and intron length distribution between the two haploid genomes of *P. trichocarpa*. Multiple protein databases were used for the comparative analysis of the predicted proteins in both haplotypes. Of the 35 979 proteins predicted in Ptr_A, 35 582 matched with the NR database and received at least one annotation. The Swissprot database annotated 28 408 proteins; the Gene Ontology (GO) database annotated 18 722 proteins; the Kyoto Encyclopedia of Genes and Genomes (KEGG) database annotated 16 890 proteins; and the Pfam database annotated 30 930 proteins. For Ptr_B, of the 36 192 predicted proteins, 35 697 matched with the NR database. The Swissprot database annotated 28 418 proteins; the GO database annotated 18 696 proteins; the KEGG database annotated 16 883 proteins; and the Pfam database annotated 30 939 proteins (Fig. 2A and Table 4).

**Table 4:** Genome annotation statistics.

**Parameter**	**Ptr_A**	**Ptr_B**
Number of annotated genes	30 089	30 331
Number of annotated mRNAs	35 979	36 192
Number of annotated exons	207 045	207 726
Number of annotated introns	171 066	173 534
Average length of mRNAs (bp)	3375.7	3362.5
Average length of exons (bp)	234.2	234.1
Average length of introns (bp)	426.5	426
Number of proteins annotated by NR	35 582	35 697
Number of proteins annotated by Swissprot	28 408	28 418
Number of proteins annotated by GO	18 722	18 696
Number of proteins annotated by KEGG	16 890	16 883
Number of proteins annotated by Pfam	30 930	30 939

### Comparative genomic and phylogeny analysis

Collinearity analysis using JCVI between the Ptr_A, Ptr_B, and the reference genome Ptr v4.1 revealed highly conserved syntenic relationships (Fig. 2C), indicating significant sequence and structural integrity maintained throughout their evolutionary history. Using SyRI, we performed whole-genome comparative analysis between these genomes (Supplemental Fig. S21). Comparison between Ptr_A and the reference genome Ptr v4.1 revealed approximately 368 Mb of syntenic regions with 1 586 306 SNPs and 406 443 InDels (comprising 6.13 Mb) (Supplemental Table S7). Similarly, between Ptr_B and Ptr v4.1, we identified approximately 366 Mb of syntenic regions, containing 1 631 616 SNPs and 433 446 InDels (comprising 5.83 Mb) (Supplemental Table S8). The comparison between haplotypes Ptr_A and Ptr_B identified approximately 362 Mb of syntenic regions. Within these syntenic regions, Ptr_A contained 3 343 223 SNPs and 779 622 InDels, spanning a total of 10.78 Mb. Similarly, Ptr_B harbored 3 339 802 SNPs and 779 845 InDels, covering a total of 10.57 Mb (Supplemental Table S9). We also conducted comprehensive synteny analysis among Ptr_A, Ptr_B, and a doubled haploid line (DH15) derived from *Populus ussuriensis* (Supplemental Fig. S22 and Supplemental Tables S10 and S11).

We conducted a quantitative analysis of the distribution of single nucleotide polymorphisms (SNPs) across different genomic regions in the haplotype genomes Ptr_A and Ptr_B. The results revealed that out of the 3 343 223 SNPs in Ptr_A, 94 106 (2.81%) were located in exonic regions, 240 345 (7.19%) in intronic regions, 25 292 (0.76%) in genic regions (nonexonic/intronic), and 2 983 480 (89.24%) in intergenic regions. Similarly, among the 3 339 802 SNPs in Ptr_B, 94 340 (2.82%) were situated in exonic regions, 242 749 (7.27%) in intronic regions, 26 816 (0.80%) in genic regions (nonexonic/intronic), and 2 975 897 (89.10%) in intergenic regions. These findings demonstrate that the distribution patterns of SNPs are highly similar between the two haplotype genomes, with the majority of SNPs (approximately 89%) located in intergenic regions with a density of about 10 SNPs/Kb, while SNPs residing in genic regions (exons, introns, and other genic regions) account for only about 11% of the total with a lower density of approximately 3.7 SNPs/Kb.

We performed comprehensive functional annotation of SNPs in the Ptr_A and Ptr_B genomes using SnpEff. According to the SnpEff classification criteria, SNPs can be divided into four major categories: high impact, moderate impact, low impact, and modifier. In the Ptr_A genome, we identified a total of 3 343 223 SNPs, including 500 high-impact SNPs (0.015%), 50 404 moderate-impact SNPs (1.507%), and 43 074 low-impact SNPs (1.288%), with the remaining 3 249 245 classified as modifiers (97.18%). Similarly, in the Ptr_B genome, we found 3 339 802 SNPs, comprising 459 high-impact SNPs (0.014%), 50 676 moderate-impact SNPs (1.517%), and 42 944 low-impact SNPs (1.285%), with the rest 3 245 723 being modifiers (97.184%) (Supplemental Table S12). The high-impact SNPs include nonsense mutations, loss of start codon, and splice site alterations, which can lead to the loss of protein function. The moderate-impact SNPs involve amino acid changes, in-frame insertions/deletions, and splice region variants. The low-impact SNPs include synonymous variants, 5′/3′ untranslated region variants, and intron variants. In addition to generating SNP density plots for Ptr_A and Ptr_B chromosomes using Ptr v4.1 as the reference genome, we also constructed chromosomal SNP density plots between these two haplotype genomes to further investigate their differences (Supplemental Figs S24–S27). The identification of these functional SNPs provides an important foundation for subsequent molecular mechanism studies and genetic improvement efforts (Supplemental File).

Thirteen genomes, including Ptr_A and Ptr_B, were used for homologous gene identification and gene family analysis: *S. dunnii* [24]*, S. brachista* [25]*, S. suchowensis* [26]*, S. viminalis* [27]*, P. euphratica* [28]*, P. ilicifolia* [29]*, P. alba* [30]*, P. tomentosa* [7]*, P. wilsonii* [31]*, P. nigra*, *and P. deltoides* [5]*.*

**Figure 3:**
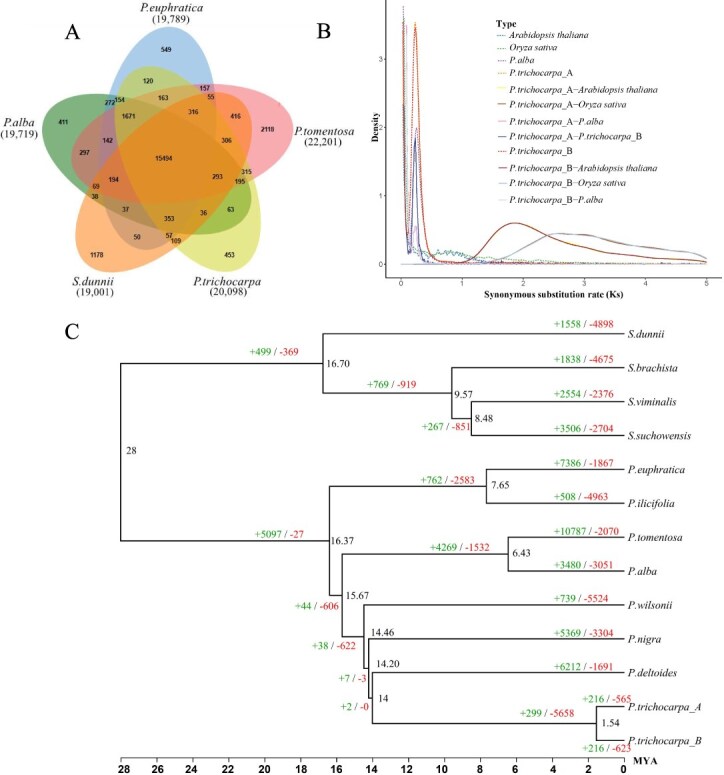
Evolutionary analysis of the *P. trichocarpa* haplotype genomes. (A) Venn diagram of gene family clustering, with numbers representing the quantity of gene families. (B) Ks distribution diagram. (C) Phylogenetic tree of *S. dunnii*, *S. brachista*, *S. suchowensis, S. viminalis, P. euphratica, P. ilicifolia, P. alba, P. tomentosa, P. wilsonii, P. nigra,* and *P. deltoides*

A phylogenetic tree was constructed based on the single-copy orthologous genes (Fig. 3C). Simultaneously, whole-genome duplication events were analyzed for these 13 species to understand their evolutionary history. A total of 31 086 gene families, comprising 519 965 genes, were identified across all species, including 204 single-copy gene families (Supplemental Table S6).

For evolutionary divergence analysis, we calculated the synonymous substitution rates (Ks) between *P. trichocarpa* haplotypes (Ptr_A and Ptr_B) and selected reference species (*Arabidopsis thaliana*, *Oryza sativa*, and *P. alba*). Genomic analysis revealed that Ptr_A and Ptr_B contain 30 089 and 30 331 genes, which can be classified into 18 313 and 18 282 gene families, respectively. Through comparative genomic analysis, we identified both species-specific and shared gene families among the five species. Notably, 10 304 conserved gene families were found to be shared among all five species, while 304 and 309 gene families were unique to Ptr_A and Ptr_B, respectively, indicating their distinct evolutionary characteristics (Fig. 3A).

The Ks distribution plot reveals critical insights into the evolutionary history of these species. High-density peaks at lower Ks values (0–0.5) were observed within Populus species, including both *P. trichocarpa* haplotypes and *P. alba*, suggesting recent divergence events. When comparing *P. trichocarpa* with *Oryza sativa*, the analysis revealed distinct peaks in the range of 1.5–2.5, indicating ancient divergence events. The distribution patterns further extend into higher Ks values (2.5–5.0), particularly evident in the comparisons between *P. trichocarpa* and *Oryza sativa*, reflecting the deep evolutionary divergence between these distant plant lineages. These patterns provide important insights into the evolutionary history and divergence times among these species (Fig. 3B).

### ASE in different tissues

**Figure 4:**
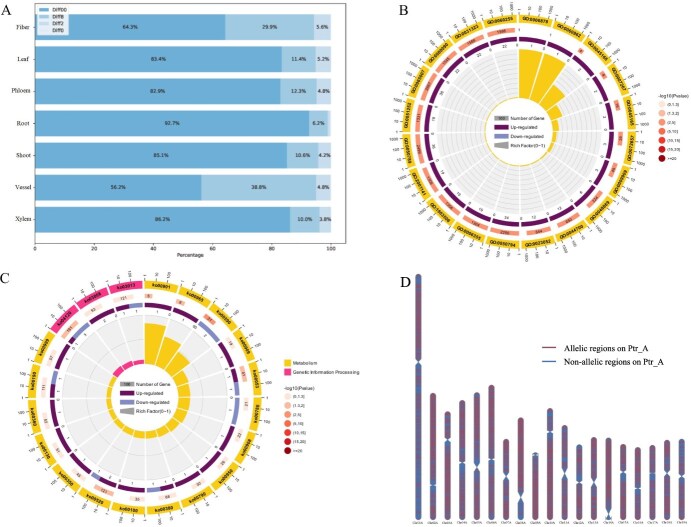
ASE analysis in seven different tissues of *P. trichocarpa*: fiber, leaf, phloem, root, shoot, vessel, and xylem. (A) The proportion of different ASE in the seven tissues of *P. trichocarpa*. (B) GO enrichment circle plot of the root tissue. (C) KEGG enrichment circle plot of the root tissue. (D) Distribution of allelic genes in the root tissue of Ptr_A chromosomes

RNA-seq data from seven tissues (fiber, leaf, phloem, root, shoot, vessel, and xylem) of *P. trichocarpa* were used alongside the Ptr_A and Ptr_B genomes to identify 21 635, 24 384, 24 150, 23 372, 24 718, 19 580, and 24 804 allele pairs in each tissue, respectively. ASE was classified into two major categories: no significant difference (Diff00) and significant ASE. The significant ASE group was further divided into three subcategories based on the fold change (FC) in expression: (1) Diff0, when FC ≤ |2|; (2) Diff2, when |2| < FC < |8|; and (3) Diff8, when FC ≥ |8|. Specifically, in the DESeq2 results, if the adjusted *P*-value (padj) of the allele was ≥0.05, it was defined as ‘no significant difference’ (Diff00). If padj ≤0.05, it was defined as ‘significant ASE’, with further classification into Diff0, Diff2, and Diff8 based on the FC (Fig. 4A). We noted that 17 148 allele pairs were commonly present across all seven examined tissues, suggesting their potential involvement in fundamental physiological processes in plants. Meanwhile, we identified tissue-specific allele pairs, with 180, 96, 64, 132, 77, 136, and 161 pairs specifically expressed in fiber, leaf, phloem, root, shoot, vessel, and xylem tissues, respectively. This coexistence of conserved and tissue-specific expression patterns reveals the sophisticated regulatory mechanisms evolved in *P. trichocarpa*, which maintain both the stability of basic physiological functions and the implementation of tissue-specific functions (Supplemental Fig. S28). Furthermore, we performed a visualization analysis of allelic genes in the root tissue of Ptr_A haplotype genome, while the visualization profiles for allelic genes in other tissues are presented in Supplemental Fig. S29.

Analysis of tissue-specific ASE patterns revealed distinct characteristics across different tissues. In fiber tissue, while 64.3% of alleles showed no significant difference (Diff00), a substantial proportion (29.9%) exhibited strong allelic bias (Diff8), with 5.6% showing moderate to slight bias (Diff2 and Diff0 combined). This marked allelic bias suggests strict regulation of specific genes involved in fiber development and structural maintenance.

Leaf and phloem tissues displayed similar expression patterns, with high proportions of Diff00 (83.4% and 82.9%, respectively) and relatively small proportions of biased expression (Diff2: 11.4% and 12.3%; Diff0: 5.2% and 4.8%). This predominantly balanced expression likely supports their roles in photosynthesis and metabolite transport.

Root tissue exhibited the highest proportion of balanced expression (Diff00: 92.7%) among all tissues, with only 6.2% showing moderate to slight bias, indicating highly stable gene expression patterns essential for water and nutrient absorption. Similarly, Shoot tissue maintained a high proportion of Diff00 (85.1%), with 10.6% moderate bias and 4.2% slight bias, reflecting the need for balanced expression in developing tissues.

Vessel tissue showed a distinctive pattern with the lowest proportion of Diff00 (56.2%) and a notably high proportion of Diff8 (38.8%), second only to fiber tissue. This strong allelic bias suggests precise regulation of genes involved in vascular development and function. Xylem tissue maintained relatively balanced expression (Diff00: 86.2%), with moderate (10.0%) and slight (3.8%) bias, supporting its role in structural support and water transport.

These tissue-specific ASE patterns reflect the specialized functions and biological requirements of different plant tissues. The high proportion of strong allelic bias (Diff8) in fiber and vessel tissues suggests precise regulation of structural and transport-related genes, while the predominantly balanced expression in root, shoot, and xylem tissues indicates the importance of stable gene expression in maintaining basic physiological functions.

### GO and KEGG enrichment analysis

GO and KEGG enrichment analyses were performed on differentially expressed alleles identified in seven tissues of *P. trichocarpa* to understand their functional characteristics (Supplemental Figs S17 and S18). The analyses revealed tissue-specific patterns in biological processes, molecular functions, and metabolic pathways.

In molecular function, fibers and phloem were notably enriched in transcription factor activities, including ‘nucleic acid binding transcription factor activity’, ‘transcription factor activity’ and ‘sequence-specific DNA binding’. This suggests an active involvement in transcriptional regulation, critical for their functional roles. Conversely, leaves and roots showed strong enrichment in enzyme inhibitor activities, likely linked to their roles in plant defense and hormonal regulation (Fig. 4B).

The phloem also displayed enrichment in specialized functions such as sulfotransferase and chitinase activities, highlighting its unique metabolic processes. Across tissues, transcriptional regulation was predominant, especially in fibers, vessels, and xylem, where it likely plays a vital role in their structural and functional integrity.

**Figure 5:**
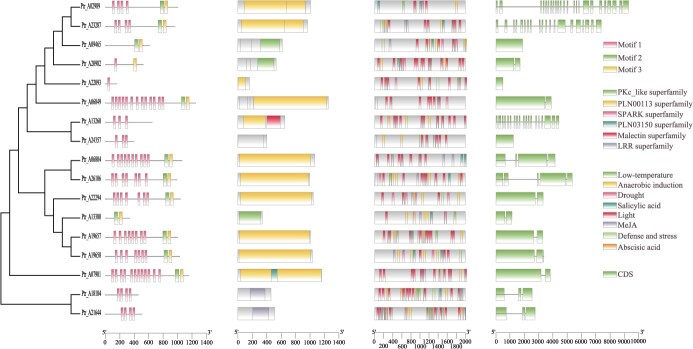
LRR gene family in the *P. trichocarpa* haplotype genome. From left to right, the phylogenetic tree of the LRR gene family, identified motifs of the LRR gene family, statistics of cis-elements in the promoter regions of LRR genes, and the structure of LRR genes

Cellular component analysis revealed differential enrichment: leaves and shoots were associated with cellular secretion and material transport, while vessels showed a specific enrichment in the NAD(P)H dehydrogenase complex, highlighting their specialized energy metabolism functions.

KEGG pathway analysis corroborated these findings, showing active involvement in metabolic and biosynthetic pathways across all tissues, essential for growth and development (Fig. 4C and Supplemental Fig. S19). Specific pathways like phenylpropanoids in fibers and pigment biosynthesis pathways in leaves and shoots were notably enriched, reflecting their specialized functions. Furthermore, pathways related to genetic information processing and environmental information processing, such as ‘Ribosome’ and ‘Plant hormone signal transduction’ were prevalent, emphasizing the sophisticated regulatory mechanisms essential for developmental processes.

These results contribute significantly to our understanding of the functional specialization and complex regulatory networks in *P. trichocarpa*, providing valuable insights for further research into tissue-specific gene functions and adaptations.

### Analysis of LRR gene family

The LRR gene family plays a pivotal role in the plant immune system. By encoding proteins with LRR sequences, these genes are integral to the recognition and defense response against pathogens. During this process, LRR proteins detect specific molecular patterns on pathogen surfaces, triggering a cascade of signal transduction pathways that activate plant defense mechanisms.

In this study, we targeted the LRR gene family for in-depth analysis. Using both HMM and BLAST methods, we identified 191 high-confidence LRR gene family members in Ptr_A. We focused on 17 LRR gene family members that exhibited ASE in the leaf tissue for further detailed analysis (Fig. 5). By utilizing the PlantCARE database for promoter region analysis of the LRR gene family, we identified the following key cis-elements and their potential regulatory mechanisms.

Low-temperature responsive element (LTR) regulates gene expression under low temperature conditions, aiding plants in adapting to cold environments. Anaerobic responsive element (ARE) activates gene expression under anaerobic conditions, helping plants survive in waterlogged or oxygen-deficient environments. MYB binding site involved in drought-inducibility (MBS) responds to drought stress, regulating gene expression to enhance drought tolerance. TCA-element is associated with the salicylic acid signaling pathway, regulating defense gene expression to enhance disease resistance. G-box is involved in light signal transduction, regulating light-responsive gene expression, thus influencing photosynthesis and plant development. CGTCA-motif responds to methyl jasmonate signaling, regulating gene expression to enhance resistance to pests and diseases. W-box is recognized by WRKY transcription factors, regulating genes involved in defense and stress responses. Abscisic acid responsive element (ABRE) responds to abscisic acid, regulating gene expression to cope with various environmental stresses such as drought and salt stress.

The expression of the LRR gene family is particularly critical under specific environmental conditions or developmental stages. For instance, under low temperature and drought conditions, LRR genes are regulated by LTR and MBS elements, helping plants adapt and resist these adverse environments. In anaerobic conditions, ARE elements enhance plant survival in waterlogged or other oxygen-deficient environments. Under pest and disease pressure, TCA-element and CGTCA-motif regulate defense responses, while G-box elements influence photosynthesis and growth during light fluctuations.

Notably, the LRR gene family plays a central role in immune regulation in plants. These genes encode proteins with LRR sequences, which are crucial for recognizing pathogenic attacks. LRR proteins activate plant defense mechanisms by identifying specific molecular patterns from external pathogens, significantly contributing to the plant's ability to adapt to environmental pressures. The mechanisms of evolution and variation accumulation in the LRR gene family vary significantly among plants with different generational times. For plants with short life cycles and rapid generational turnover, natural and artificial selection can quickly promote the adaptive evolution of LRR genes. In perennial plants, such as poplars, this process is slower, making it challenging to improve their LRR gene composition and function through traditional methods.

Moreover, the expansion and diversity of the LRR gene family are largely attributed to tandem duplication events, which can rapidly generate a large number of gene copies, increasing genetic diversity. By analyzing the LRR gene family, we can gain a deeper understanding of how plants utilize gene family expansion to cope with biotic stresses in the environment.

Considering the characteristics of perennial plants like poplars, we particularly focused on the genetic diversity and heterosis introduced through hybridization, which is crucial for enhancing their biological adaptability. The analysis of the LRR gene family, combined with ASE analysis, can reveal which gene expression variations in hybrids are particularly effective in enhancing plant resistance. This approach provides a potential strategy to increase beneficial variations in the LRR gene family through scientific breeding methods, further enhancing the biological defense capabilities of poplars.

## Discussion

*P. trichocarpa*, also known as black cottonwood, is an important species in the genus Populus of the Salicaceae family. It was the first tree species to have its genome fully sequenced and is a model species for woody plant transgenics. The wood fibers of *P. trichocarpa* are high-quality raw materials for papermaking. It has a high biomass and short growth cycle, making it an ideal raw material for biomass energy. Its bark contains various medicinal components, such as salicin and flavonoids. In previous studies, the *P. trichocarpa* genome was assembled as a ‘mosaic’ haploid model, and no chromosomal-scale and haplotype-resolved genome has been released to date. This study presents a new version of the near T2T assembled haplotype-resolved *P. trichocarpa* genome.

In this study, the first near T2T (containing very few gaps and identifying the vast majority of telomere and centromeric regions) haplotype-resolved *P. trichocarpa* genomes were constructed. The genome sizes of Ptr_A and haplotype Ptr_B are 391.76 and 397.43 Mb, respectively, which are close to the 392.16 Mb of the *P. trichocarpa* v4.1 ‘mosaic’ genome. These differences may arise from advancements in sequencing technology, redundancy in the ‘mosaic’ genome assembly, and gap filling in the near T2T assembly. In this study, 30 089 and 30 331 genes were annotated in the Ptr_A and Ptr_B genomes, respectively, and the annotation results were obtained under strict conditions to remove false positives. Through genome collinearity analysis between Ptr v4.1 and the Ptr_A/Ptr_B haplotype genomes, we observed notable size variations in chromosome 14 (Supplemental Figs S20 and S21). Specifically, chromosome 14 in Ptr v4.1 exhibits an intermediate length, being slightly longer than Chr14A but shorter than Chr14B. To validate the assembly accuracy of Chr14A and Chr14B, we employed the recently developed genome continuity inspector (GCI) tool [32], which incorporates multiple alignment algorithms for quantitative assessment of assembly quality. The evaluation results (Supplemental Fig. S23) demonstrated exceptional assembly quality for both Chr14A and Chr14B, with quality scores exceeding 99 on a 100-point scale, thus confirming the high reliability of our assembly. These results suggest that the observed size differences between chromosome 14 in Ptr v4.1 and its counterparts in Ptr_A/Ptr_B likely reflect genuine structural variations between the heterozygous and haplotype-resolved genomes, rather than assembly artifacts.

To investigate the genetic variation characteristics between the two haploid genomes Ptr_A and Ptr_B of *Populus trichocarpa* and the reference genome *P. trichocarpa* v4.1 from the Phytozome database, we adopted a pairwise collinearity comparison approach. Specifically, we used the SyRI tool to perform collinearity analysis and leveraged SnpEff to functionally annotate the detected SNPs, systematically evaluating the biological effects and potential functional impacts of these variant loci. This pairwise comparison strategy can precisely depict the variation landscape between the two haploid genomes of *P. trichocarpa*, and the comparison with the reference *P. trichocarpa* genome can map the variant sites to the reference genome. By in-depth analysis of the variant regions, we not only can develop molecular markers specific to the *P. trichocarpa* haplotypes, but also explore the haplotype-specific adaptive evolutionary strategies. Additionally, we have compiled the allelic variation information in the collinear regions between the two haploid genomes of *P. trichocarpa*. On the one hand, this can improve the precision of gene cloning for researchers and reduce the risk of mistaking highly variable alleles as new genes; on the other hand, it can also enable forestry research to embark on studies related to allelic functional differences and utilization, similar to the progress in agriculture. These efforts are of great significance for deeply understanding the mechanisms of heterosis and phenotypic variation in long-lived woody plants, and promoting the development of molecular biology and breeding in forestry.

In our haploid genomes Ptr_A and Ptr_B, there are identified 6 and 2 gaps, respectively. Inspired by recent research on the T2T genome of poplar by [33], we conducted annotation analysis on sequences not assigned to any chromosome. We discovered 67 rRNA clusters in the remaining contigs not mounted to Ptr_A, and 46 rRNA clusters in those not mounted to Ptr_B. These findings support the hypothesis that gap regions are predominantly composed of rDNA, and that the length of HiFi reads is insufficient to traverse the repetitive regions of rRNA clusters. Additionally, rRNA clusters were detected on chromosome 8 of Ptr_A, and on chromosomes 8 and 14 of Ptr_B. All results regarding the rRNA clusters are detailed in the Supplemental files.

After assembling the near T2T haplotype genome of *P. trichocarpa*, we conducted ASE analysis using RNA-seq data [34]. This analysis allowed us to study the different expression patterns of alleles in the seven tissues and investigate potential biological functional differences. For diploid species, traditional ‘mosaic’ genome analysis may lead to inaccurate allele expression analysis due to the presence of mosaic regions. The use of haplotype genomes avoids this complexity and accurately reflects the true expression levels of each allele.

The LRR gene family, which has been well studied in model plants such as rice and Arabidopsis, is involved in various stress responses and biological processes, playing a key role in pathogen recognition, defense responses, and the overall immune system. In this study, 191 members of the LRR gene family were identified in the *P. trichocarpa* haplotype genome (Ptr_A), and further analysis revealed that these genes were widely expressed in seven tissues of *P. trichocarpa*. The cis-elements identified in the promoter regions of *P. trichocarpa* LRR genes included elements related to light response, defense and stress responses, drought-inducibility, salicylic acid response, low-temperature response, anaerobic-specific induction, gibberellin, and auxin response, indicating that LRR family genes are important regulatory factors for adaptability and physiological functions in *P. trichocarpa*.

This study of the LRR gene family in *P. trichocarpa* provides a theoretical basis for further functional research, gene editing, and improvement of plant varieties.

## Materials and methods

### Plant materials

The sequencing material comprising axenic *P. trichocarpa* Nisqually-1 plantlets was provided by Professor Vincent L. Chiang from the National Key Laboratory of Tree Genetics and Breeding. These are the clonal offspring of *P. trichocarpa,* which were first sequenced in 2006 [1]. Leaves were transported on dry ice to a sequencing company.

### Genome sequencing

#### Library preparation and PacBio HiFi sequencing

Quality control of DNA samples mainly involves two methods: agarose gel electrophoresis to analyze the degree of DNA degradation and detect potential RNA and protein contamination, and Qubit to accurately quantify DNA concentration. After passing the sample quality control, the genomic DNA was fragmented and the fragments were size-selected using BluePippin. After end repair and A-tailing, adapters were ligated to both ends of the fragments to prepare the DNA library. Once the library passed the quality control, sequencing was performed on the PacBio Sequel II platform based on the effective library concentration and desired data output. SMRTlink software was used to preprocess the raw sequencing data, and the ccs command was employed for HiFi analysis. The data preprocessing mainly includes the following steps: adapter removal, from the polymerase reads obtained by single-molecule sequencing, and splitting into subreads sequences; the subreads from the same ZMW undergo self-correction to form HiFi sequences, which are used for subsequent analysis.

#### Hi-C sequencing

Hi-C technology originates from Chromosome Conformation Capture (3C). It focuses on the entire cell nucleus and combines high-throughput sequencing technology with bioinformatics methods to investigate the spatial relations of chromatin DNA across the genome. Through capturing the interaction patterns of all DNA within chromatin, the Hi-C technique provides high-resolution information on the three-dimensional structure of chromatin.

### Genome survey

Before performing genome assembly, It is crucial to estimate the genome size, GC content, and heterozygosity for genome assembly. Jellyfish v2.3.0 [35] was used to analyze the HiFi sequencing data of *P. trichocarpa*, yielding *k*-mer distribution data. Subsequently, GenomeScope v1.0 [36] was used to assess the genome size, GC content, and heterozygosity of *P. trichocarpa* based on the *k*-mer analysis results.

### Genome assembly

Firstly, Hifiasm v0.18 [19] was used in Hi-C mode, incorporating Hi-C data, to assemble the HiFi sequencing data into haplotype contigs. During this process, parameters were modified according to the log prompts.

YaHS v1.1 [20] was employed to connect the contigs into different chromatin groups. This tool relies on a novel contig-connection detection algorithm that considers the topological distribution of Hi-C signals and reduces noise. Subsequently, JuiceBox [37] software was used for manual positioning and calibration.

The GapFiller module of quarTeT v1.1.6 [38] was utilized to fill gaps, using the manually calibrated haplotype assembly results and HiFi sequencing data as input.

### Genome annotation

#### Gene structure annotation

Gene structure annotation of the two haploid genomes utilized the braker v3.0.5 pipeline [39,40], with the latest green plant protein database Viridiplantae from OrthoDB v11 [41] serving as the source of homologous proteins for *P. trichocarpa*. Transcriptomic data from seven *P. trichocarpa* tissues were used evidence for gene structure annotations. The transcript data were aligned to the soft-masked haploid genomes using Hisat2 v2.2.1 [42]. To minimize the presence of false positives in the prediction results, we initially constructed a local repeat sequence database using the RepeatModeler tool [43] and then performed soft masking of the repetitive sequences of the genome with RepeatMasker [44], utilizing the latest TEs database Dfam.h5 [45]. The specific parameters were as follows: —genome = Ptr_A.masked.fa —prot_seq = Viridiplantae.fa —bam = ./*.sorted.bam —threads 48 —softmasking —gff3. The braker annotation process also employed the following dependency tools, BamTools v2.5.1 [46], GeneMark-ETP [47], Augustus v3.4.0 [48], Stringtie v2.2.1 [49], gffread v0.12.7 [50], and Bedtools v2.26.0 [51].

#### Repeat sequence annotation

EDTA v2.0.0 [22] was used to annotate the repetitive sequences in the haplotype genomes and generate a new TE library. The parameters used were: —genome = Ptr_A.fa —step all —cds Ptr_A.cds.fa —sensitive 1 —anno 1 —evaluate 1 -t 100.

#### Gene function annotation

The functional annotation of genes was based on the results of a previous gene structure prediction step. Based on the predicted results, translated protein sequences were extracted from the genome and aligned with protein databases including Nr [52], SWISS-PROT [53], GO [54], KEGG [55], and Pfam [56]. The alignment tool used was DIAMOND v2.0.11 [57]. This process completed functional annotation and identified biological functions and metabolic pathway information. TBtools v2.070 [58] was used to create an upset plot of functional annotation results.

#### RNA annotation

Pybarrnap v0.5.0 (https://github.com/moshi4/pybarrnap) is used for RNA annotation of genome sequences.

### ASE in different tissues

#### Alleles identification

AlleleFinder v1.0.0 (https://github.com/sc-zhang/AlleleFinder) was used to identify allelic genes in the *P. trichocarpa* haplotype genomes. The input files included the assembly results of haplotype A (Ptr_A.fasta), the annotation results of haplotype A (Ptr_A.gff3), the coding sequence file of haplotype A (Ptr_A.cds.fasta), the merged annotation results of haplotypes A and B, the merged CDS file of haplotypes A and B, and TE annotation results. The output consisted of the corresponding allelic genes for the two haplotypes. The dependent files used in this process included MCScanX [59], GMAP [60], and BLAST v2.5.0 [61].

#### ASE

Hisat2 [42] was used to build an index for haplotype genome files and align them with RNA-seq data files from different tissues to generate SAM files. SAMtools was then employed to convert the SAM files into BAM files and sort them. The processed results from the previous step were used as input for the MarkDuplicates module of GATK v4.0.5.1 [62] to remove duplicates from the BAM files, providing reliable data for the next step of analysis. Finally, the VariantFiltration module of GATK was employed to identify and filter SNPs in different tissues. VCF files containing SNPs were imported into the ASEReadCount module to calculate ReadCount values for each SNP locus. The mean ReadCount values of all SNP loci within the same allelic gene were used as the expression values.

The calculated ReadCount expression values of the allelic genes were input into DESeq2 [63] for differential expression analysis.

### GO enrichment analyses

GO enrichment analysis was performed on tissue-specific allelic genes to explore their functional differences across various tissues. The target gene set included allelic gene expression data from multiple tissues, obtained after performing rigorous quality control and differential expression analyses. The background gene set was defined using the latest version of the GO annotation database, containing all annotated genes. GO enrichment analysis was implemented using the topGO package [64] by employing Fisher's Exact Test, and the results were corrected for multiple comparisons using the False Discovery Rate method. GO terms with adjusted *P*-values less than 0.05 were selected as enriched functional categories.

### KEGG enrichment analyses

KEGG is a major public pathway database. Pathway significance enrichment analysis was performed using KEGG Pathways as the unit and applying the hypergeometric test to identify the potential roles of allelic genes in metabolic and signaling pathways. The target gene set consisted of tissue-specific allelic genes from different tissues, whereas the background gene set consisted of all genes with pathway annotations in the KEGG database. Analysis was performed using the clusterProfiler package [65] in R.

### Identification of telomeres and centromeres

Using the gap-filled FASTA file as the input, the TeloExplorer module was employed to identify telomeres in the genome. The gap-filled genome file and GFF3 file generated by the EDTA tool for TE annotation were used as inputs for the CentroMiner tool in quarTeT v1.1.6, to identify centromeres in the genome.

### Genomic evolutionary analysis

#### Phylogenetic tree construction

In addition to the two haplotypes of *P. trichocarpa* (Ptr_A and Ptr_B), other plant species were selected for genome evolution analysis: *S. dunnii*, *S. brachista*, *S. suchowensis, S. viminalis, P. euphratica, P. ilicifolia, P. alba, P. tomentosa, P. wilsonii, P. nigra, P. trichocarpa,* and *P. deltoides.* For genes with alternative splicing variants, the longest transcript was selected. Then, the comparison was performed using BLASTP with an *e*-value cut-off of 1e-5. The OrthoFinder v2.4.1 software [66] was used to perform gene family clustering of the amino acid sequences of all species. Subsequently, the species divergence times were calculated using r8s v1.81 [67] software with default parameters. The results of gene family expansion and contraction were obtained using CAFE5 [43, 68]. Finally, visualization was performed using IQ-TREE [69].

#### Genome-wide replication analysis

Based on the protein sequence alignment results from different plants in the previous step, WGD software [70] was used to perform protein collinearity analysis and analyze the distribution of synonymous mutation rates (Ks) of collinear gene pairs. Python software was used to visualize the results.

#### Collinearity analysis

JCVI v1.3.8 [71] was used to determine the regions of collinearity gene pairs in chromosomes and obtained collinear blocks. Nucmer is employed for pairwise genome alignment, followed by SyRI tool v1.7.0 [72] to identify genomic variations (including SNPs, InDels) and syntenic regions between different genomes, and plotsr v1.1.1 was used for the visualization of variations and genomic collinearity.

#### SNPs annotation

Separate reference databases were constructed for Ptr_A and Ptr_B using their respective genome assemblies, protein sequences, gene annotation files, and coding sequences. Subsequently, functional annotation analysis was performed on the SNPs identified by SyRI using SnpEff v5.2c [73] to predict the potential impacts of these variants on gene function.

### Identification and analysis of LRR gene family

To download and construct a local Pfam database, the following steps were undertaken: First, the protein sequences of the haplotype of *P. trichocarpa* were aligned against the Pfam-A.hmm using hmmscan. The results were then parsed with a Python script to filter significant matches and establish the correspondence between gene families and genes. Next, multiple sequence alignments were performed using ClustalW, followed by the construction of HMM models and a subsequent secondary search. Gene identification was carried out through BLAST comparisons to select reliable gene family members. Finally, gene IDs obtained from both HMMER and BLAST were merged, and 191 genes from the intersection were identified as high-confidence members of the LRR gene family.

The LRR family protein sequences from the *P. trichocarpa* haplotype genome were submitted to the MEME Suite website for motif information. Additionally, 2000 bp upstream sequences from the start codon (ATG) of the target genes were submitted to the PlantCARE database [74] to predict cis-acting regulatory elements (promoters) of the LRR gene family members. LRR gene family protein sequences from the haplotype genome were uploaded to the NCBI Batch CD-Search [75] to identify conserved structural domains in the genes.

## Supplementary Material

Web_Material_uhaf012

## Data Availability

The whole genome sequence data reported in this paper have been deposited in the Genome Warehouse in National Genomics Data Center, Beijing Institute of Genomics, Chinese Academy of Sciences/China National Center for Bioinformation, under accession number GWHERCM00000000 and GWHERCL00000000 that is publicly accessible at https://ngdc.cncb.ac.cn/gwh. The raw sequences are available at https://bigd.big.ac.cn/gsa/browse/CRA015670. The supplemental files can be downloaded at: http://www.wangsui.net.cn/resource/database/public/plant/Populus/trichocarpa/Supplemental_Files.zip
