# Supplementary material for: Chromosome-scale and haplotype-resolved genome assembly of Populus trichocarpa
Source: Hortic Res. 2025 Jan 15;12(4):uhaf012. doi: 10.1093/hr/uhaf012 (PMC11908830; doi:10.1093/hr/uhaf012)
Supplement: Web_Material_uhaf012 [file web_material_uhaf012.zip › Supplement Data.pdf]

Chromosome-scale and haplotype-resolved genome assembly of *Populus trichocarpa*

Gao *et al.*

Supplemental data

Supplemental Figures

Supplemental Figure S1. Quality scores across all bases of Hi-C.
Supplemental Figure S2. *K*-mer Coverage Distribution (Log Scale).
Supplemental Figure S3. *K*-mer Frequency Distribution of Ptr_A.
Supplemental Figure S4. *K*-mer Frequency Distribution of Ptr_B.
Supplemental Figure S5. Distribution of Telomeres and Gaps in Ptr_A.
Supplemental Figure S6. Distribution of Telomeres and Gaps in Ptr_B.
Supplemental Figure S7. Results of Centromeric Region Identification in Ptr_A by TBtools.
Supplemental Figure S8. Results of Centromeric Region Identification in Ptr_B by TBtools.
Supplemental Figure S9. Hi-C interaction matrix maps of 19 chromosomes in Ptr_A.
Supplemental Figure S10. Hi-C interaction matrix maps of 19 chromosomes in Ptr_B.
Supplemental Figure S11. Hi-C interaction matrix maps of Ptr_A whole genome.
Supplemental Figure S12. Hi-C interaction matrix maps of Ptr_B whole genome.
Supplemental Figure S13. Syntenic depth ratio analyses of Ptr_A and Ptr v4.1.
Supplemental Figure S14. Genome-wide syntenic relationship between Ptr_A and Ptr v4.1 assemblies (29,224 gene pairs).
Supplemental Figure S15. Syntenic depth ratio analyses of Ptr v4.1 and Ptr_B.
Supplemental Figure S16. Genome-wide syntenic relationship between Ptr_A and Ptr v4.1 assemblies (29,070 gene pairs).
Supplemental Figure S17. A Go term top 20 in issues (Fiber, Leaf, Phloem, Root, Shoot, Vessel, Xylem).
Supplemental Figure S18. Pathway Enrichment Analysis of Haplotype-Specific Alleles in *Populous trichocarpa* Using GO Database.
Supplemental Figure S19. Pathway Enrichment Analysis of Haplotype-Specific Alleles in *Populous trichocarpa* Using KEGG Database.
Supplemental Figure S20. Chromosomal Synteny Analysis between Ptr_A and Ptr_B Genomes
Supplemental Figure S21. Genome collinearity analysis between Ptr v4.1 and Ptr_A/Ptr_B haplotype genomes.
Supplemental Figure S22. Genome collinearity analysis between a doubled haploid line (DH15) of *Populus ussuriensis* and Ptr_A/Ptr_B haplotype genomes.
Supplemental Figure 23. GCI (Genome Continuity Inspector) evaluation results for Chr14A and Chr14B assemblies.
Supplemental Figure S24. SNP density of Ptr_A relative to Ptr v4.1 reference genome.
Supplemental Figure S25. SNP density of Ptr_B relative to Ptr v4.1 reference genome.
Supplemental Figure S26. SNP density of Ptr_A relative to Ptr_B reference genome.
Supplemental Figure S27. SNP density of Ptr_B relative to Ptr_A reference genome.
Supplemental Figure S28. Comparative analysis of gene expression patterns in different tissues.

Supplemental Figure S29. Distribution of allelic regions across multiple tissues in Ptr_A.

Supplemental Tables

Supplemental Table S1. Statistics of HiFi sequencing data.
Supplemental Table S2. Statistics of Hi-C sequencing data.
Supplemental Table S3. Centromeres in Ptr_B genome.
Supplemental Table S4. Busco of Ptr_A genome.
Supplemental Table S5. Busco of Ptr_B genome.
Supplemental Table S6. Overall Statistics of homologous gene identification and gene family analysis.
Supplemental Table S7. Comparative analysis between Ptr_A and the reference genome Ptr v4.1 by SyRI.
Supplemental Table S8. Comparative analysis between Ptr_B and the reference genome Ptr v4.1 by SyRI.
Supplemental Table S9. Comparative analysis between Ptr_A and Ptr_B by SyRI .
Supplemental Table S10. Comparative analysis between Ptr_A and a doubled haploid line of *Populus ussuriensis* (DH15) by SyRI.
Supplemental Table S11. Comparative analysis between Ptr_B and a doubled haploid line of *Populus ussuriensis* (DH15) by SyRI.
Supplemental Table S12. SnpEff annotation summary of SNPs of Ptr_A and Ptr_B.

Supplemental Figures

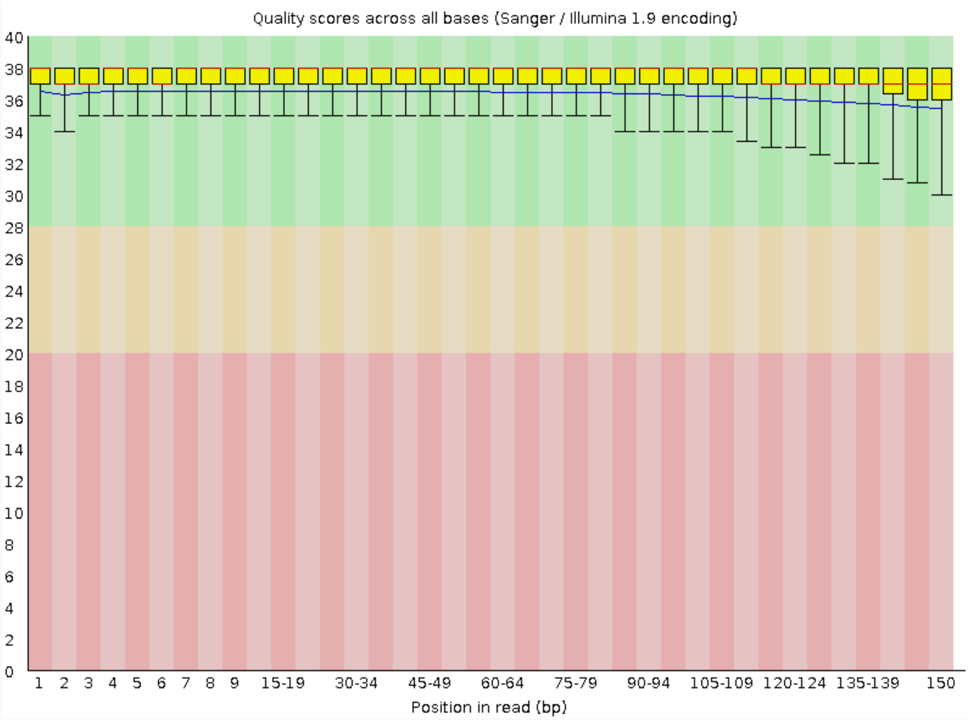

Supplemental Figure S1. Quality scores across all bases of Hi-C

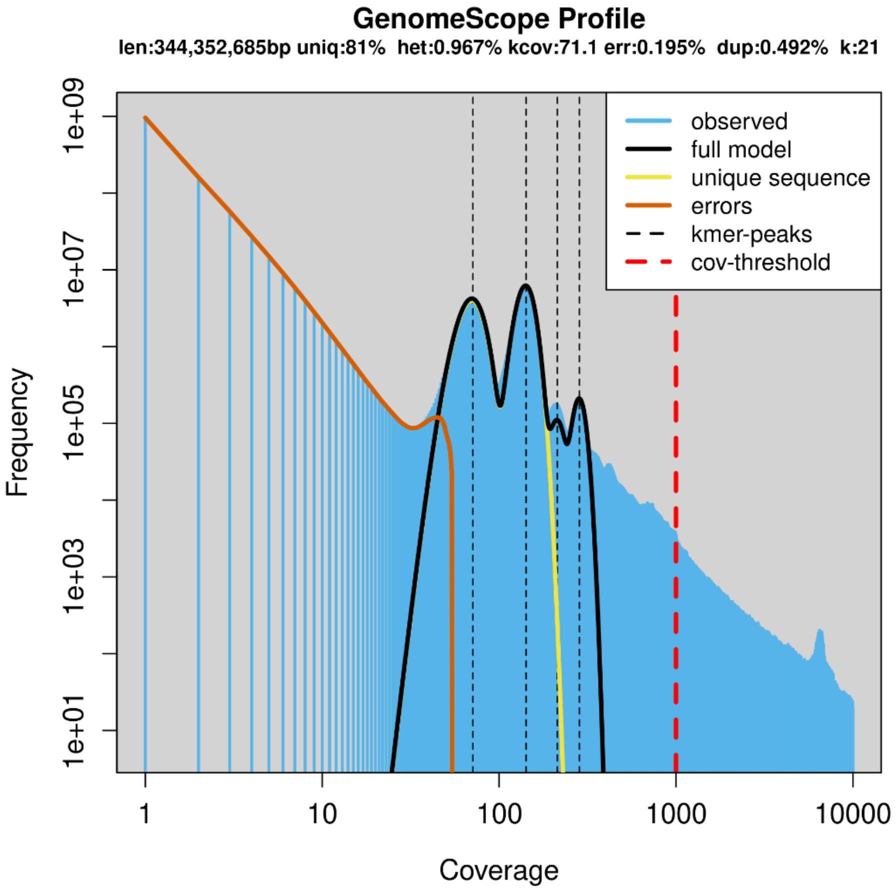

Supplemental Figure S2. K-mer Coverage Distribution (Log Scale)

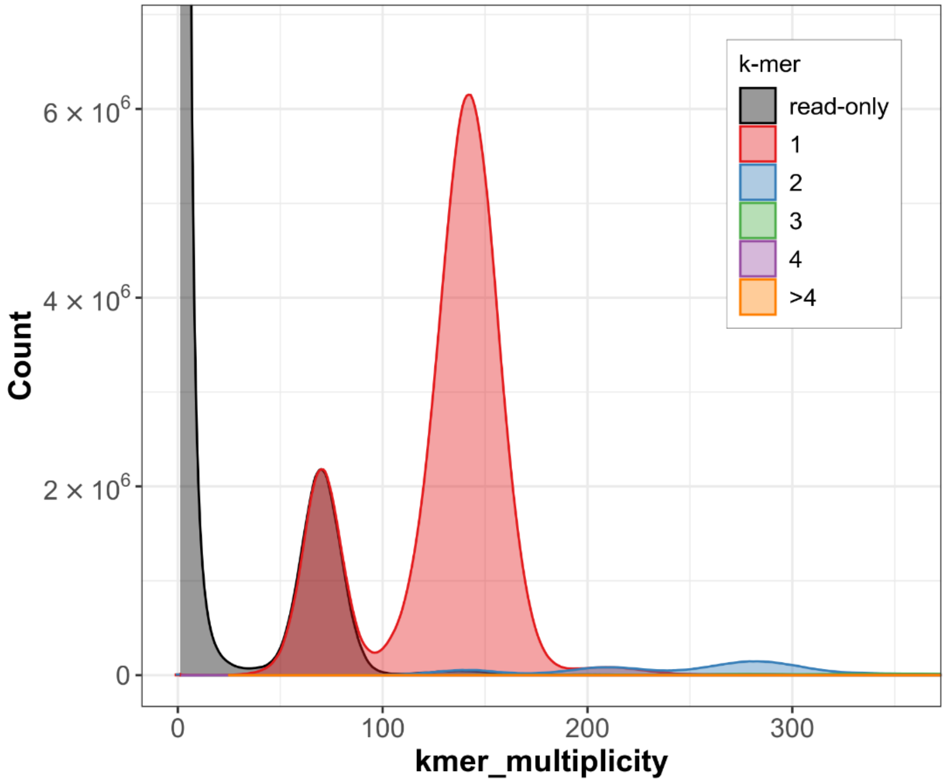

Supplemental Figure S3. K-mer Frequency Distribution of Ptr_A

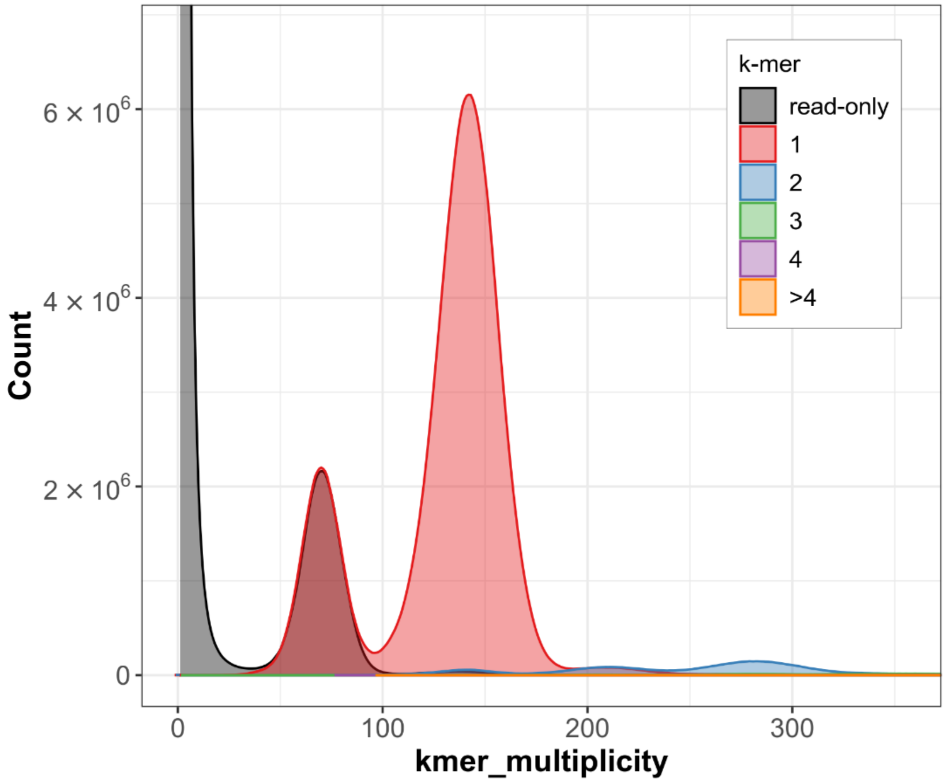

Supplemental Figure S4. K-mer Frequency Distribution of Ptr_B

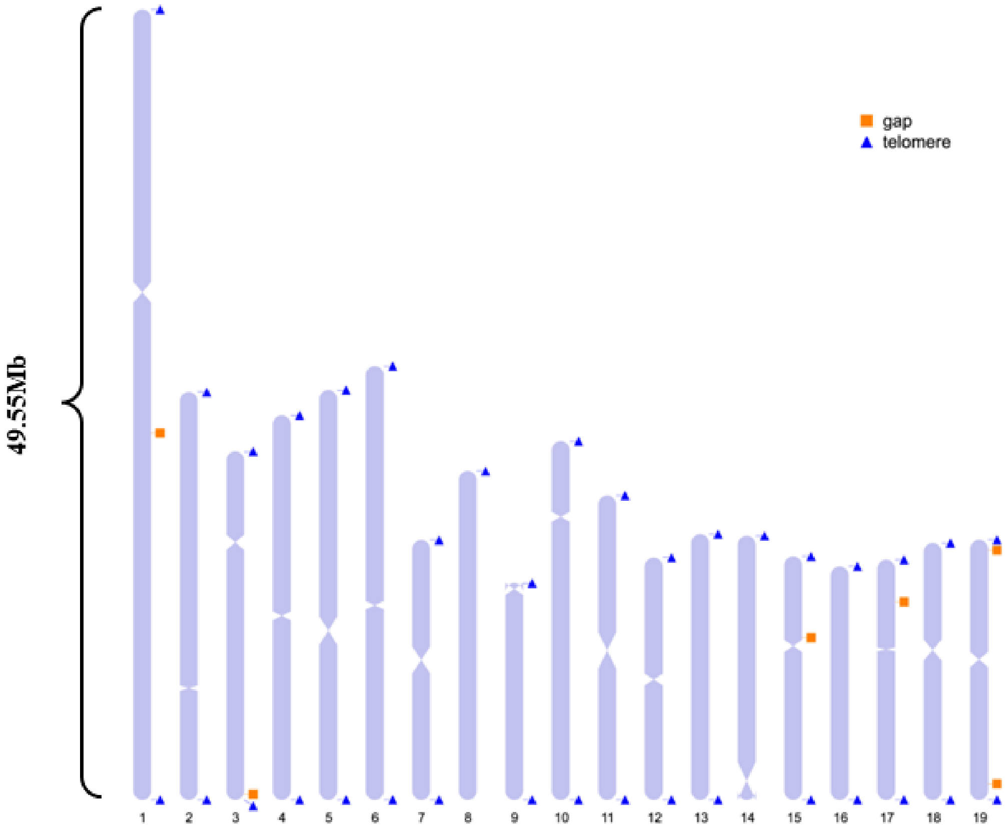

Supplemental Figure S5. Distribution of Telomeres and Gaps in Ptr_A

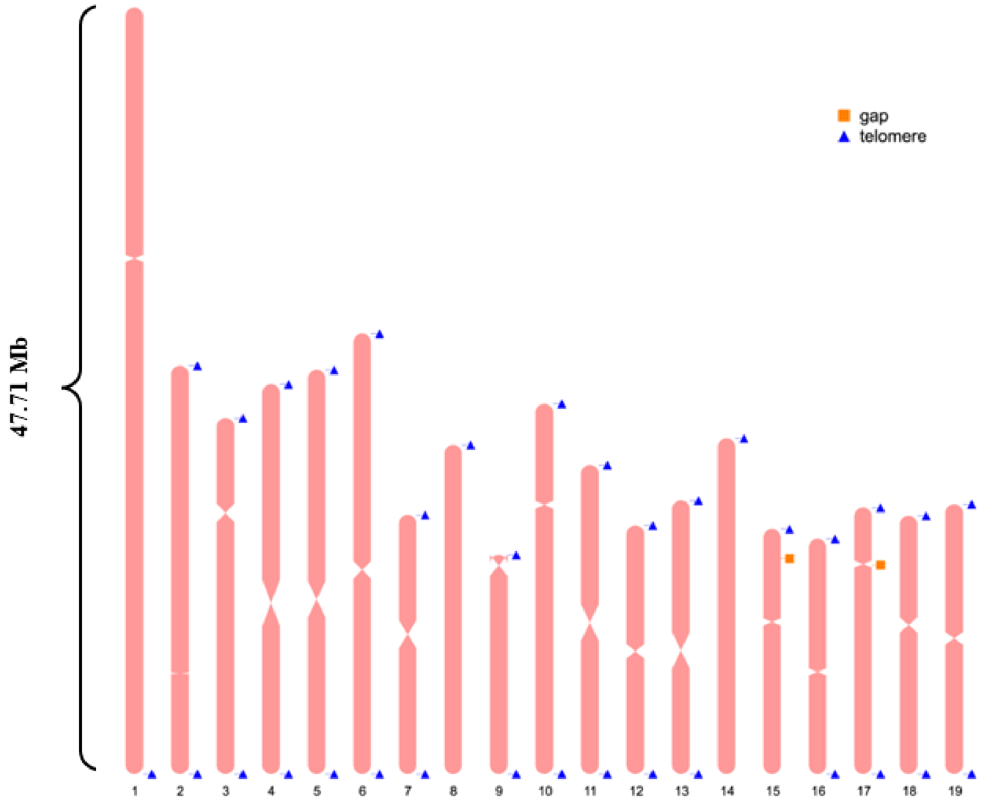

Supplemental Figure S6. Distribution of Telomeres and Gaps in Ptr_B

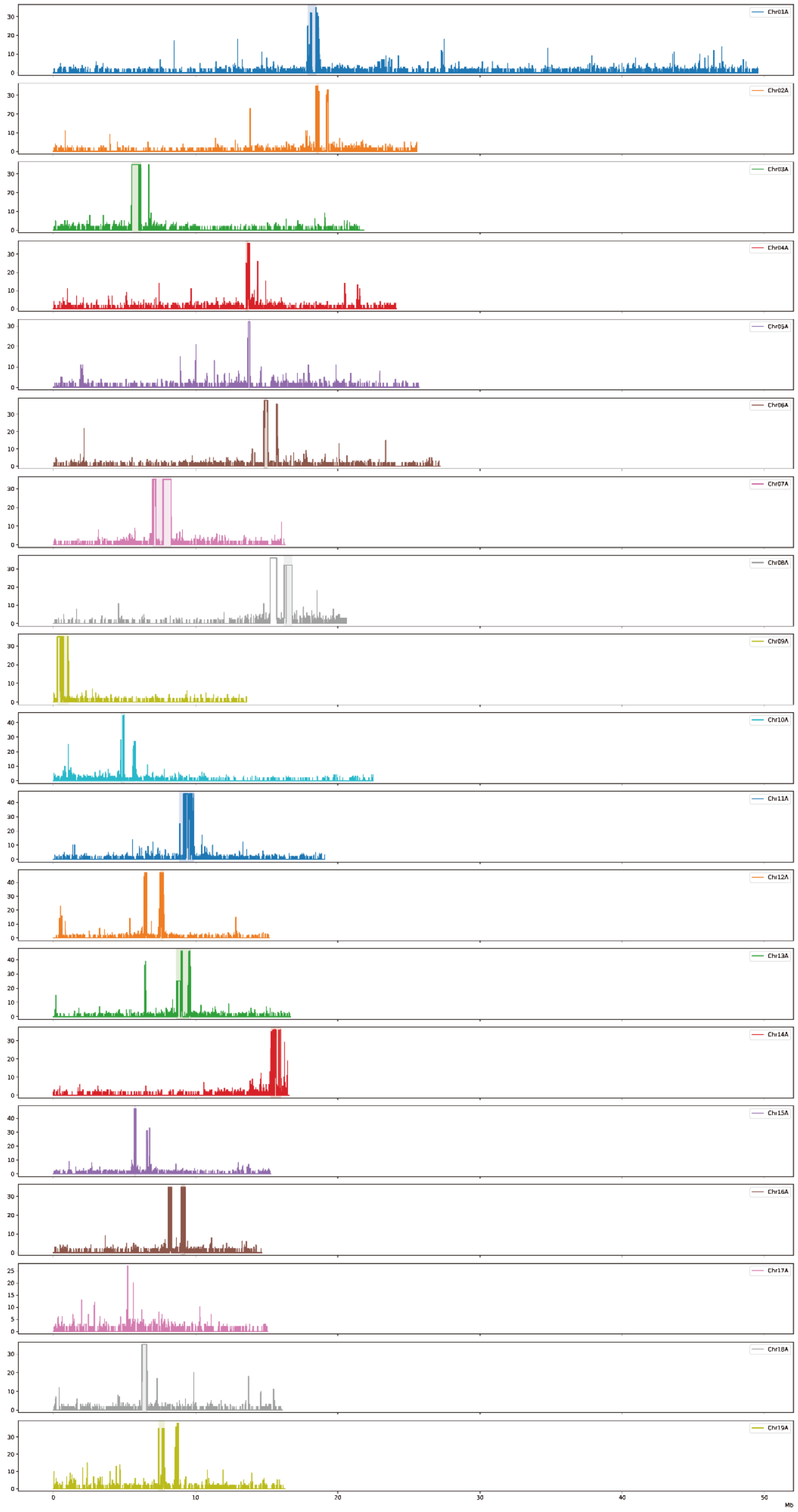

Supplemental Figure S7. Results of Centromeric Region Identification in Ptr_A by TBtools

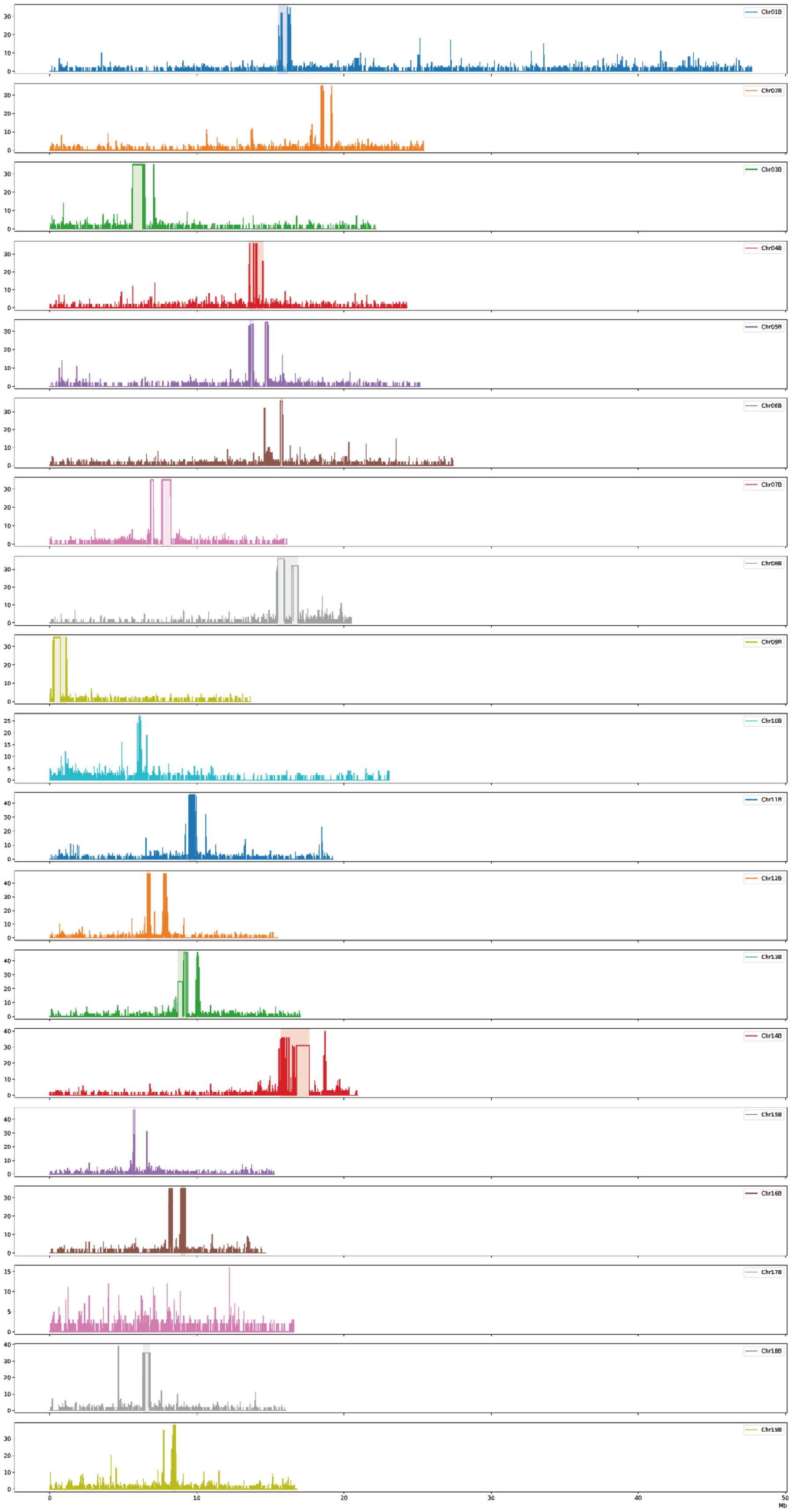

Supplemental Figure S8. Results of Centromeric Region Identification in Ptr_B by TBtools

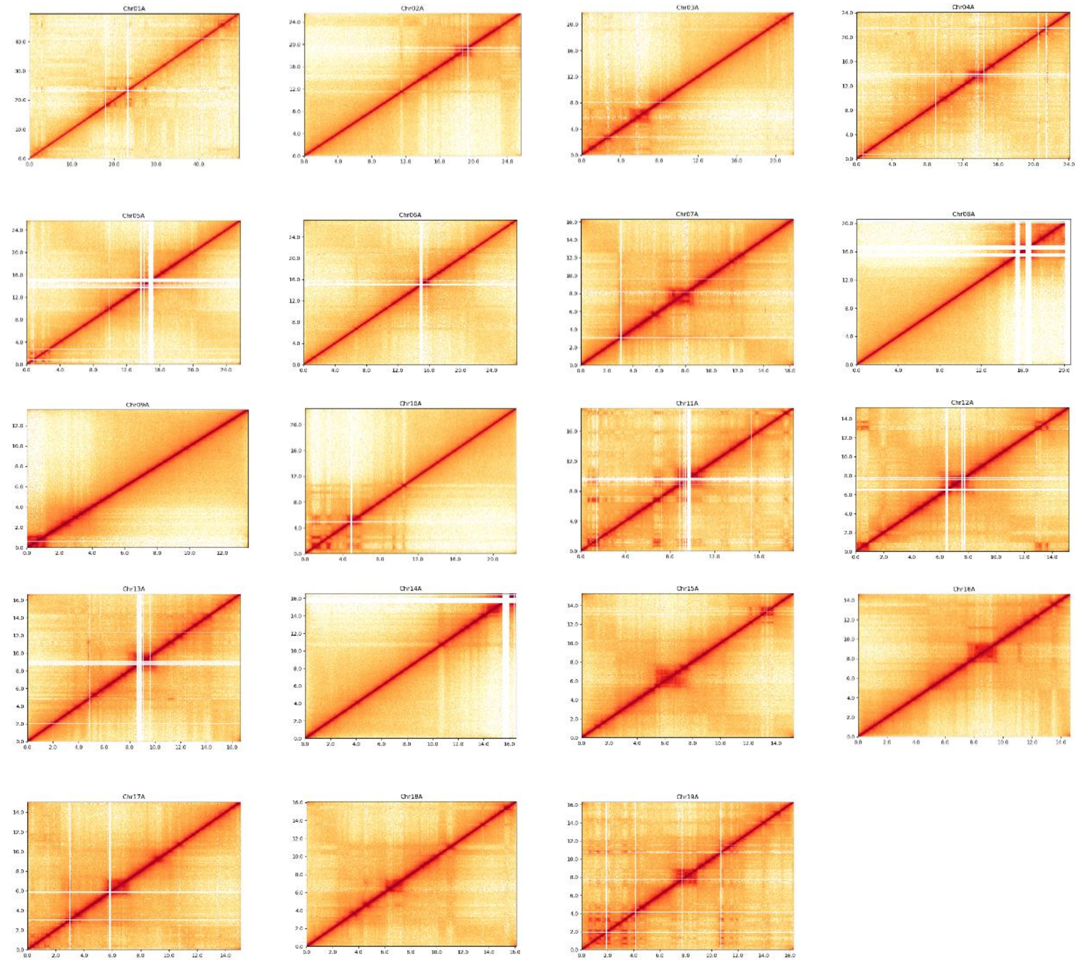

Supplemental Figure S9. Hi-C interaction matrix maps of 19 chromosomes in Ptr_A

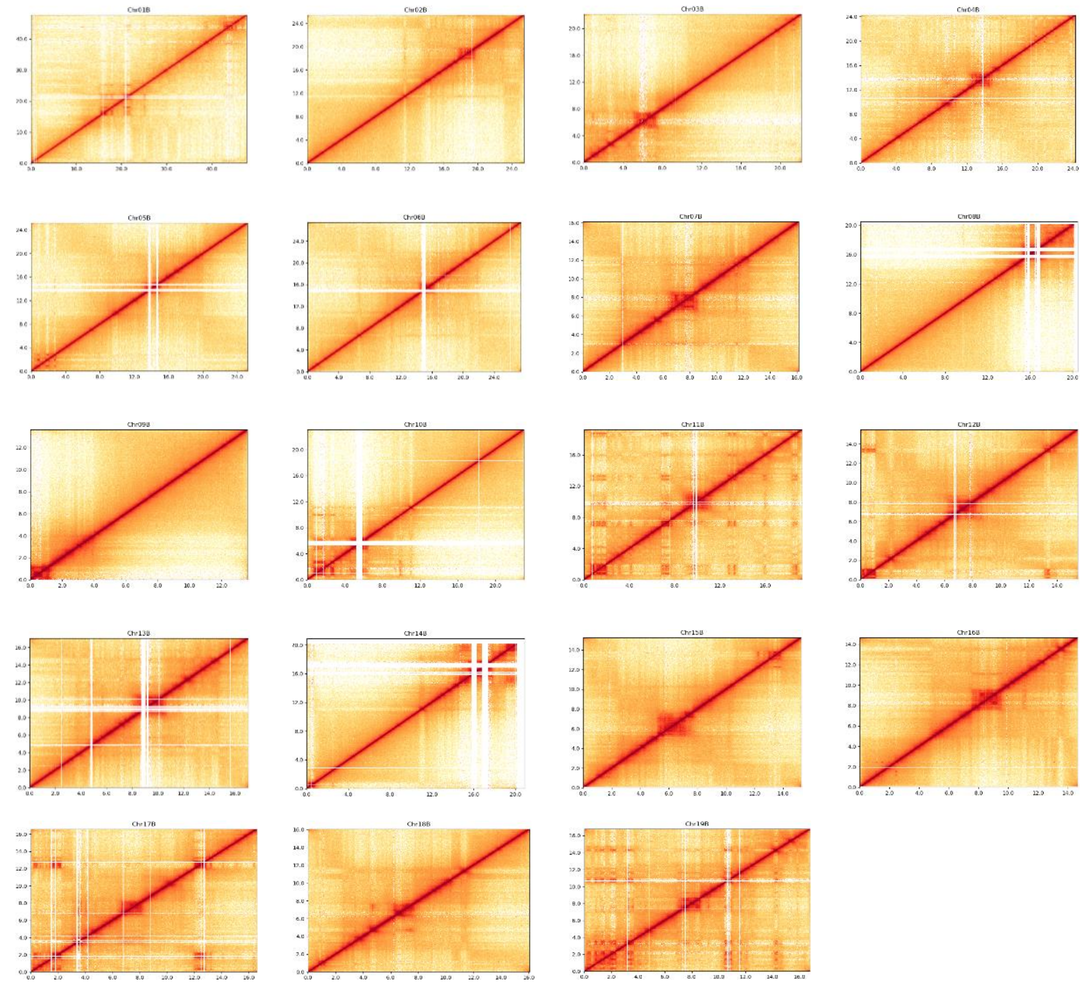

Supplemental Figure S10. Hi-C interaction matrix maps of 19 chromosomes in Ptr_B

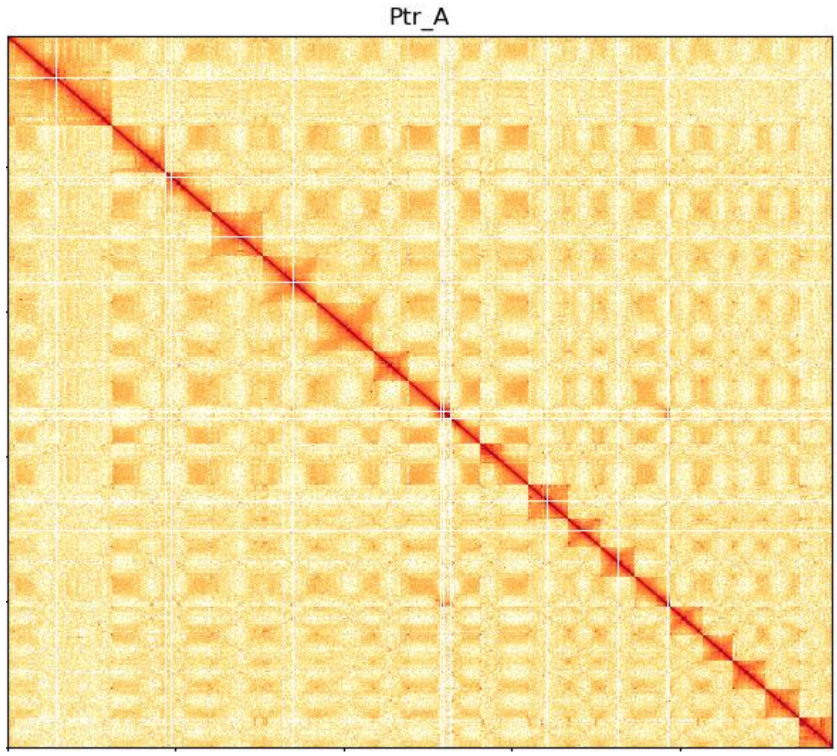

Supplemental Figure S11. Hi-C interaction matrix maps of Ptr_A whole genome

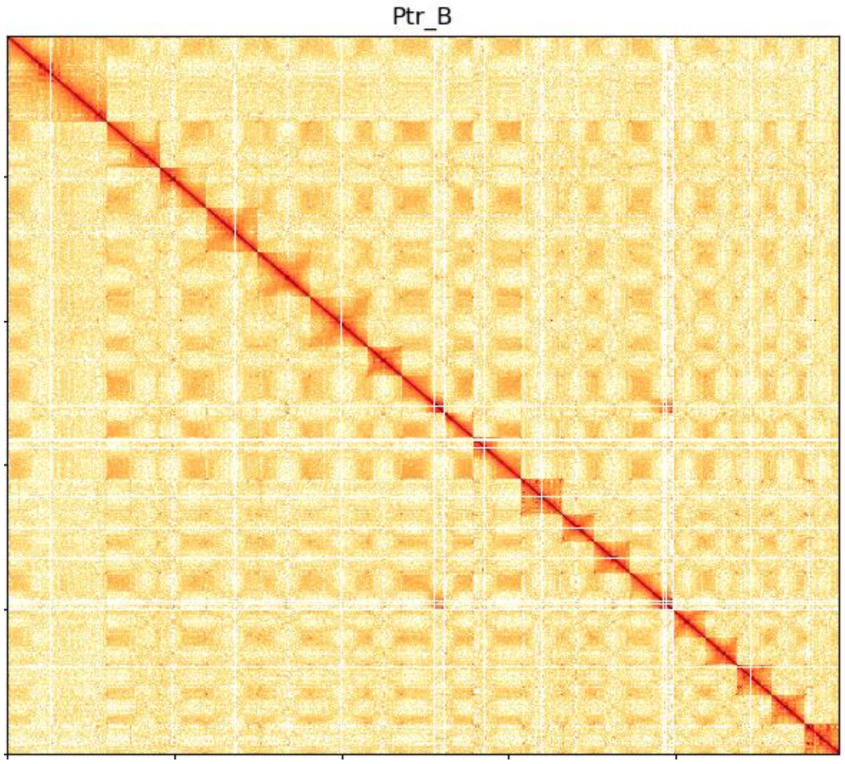

Supplemental Figure S12. Hi-C interaction matrix maps of Ptr_B whole genome

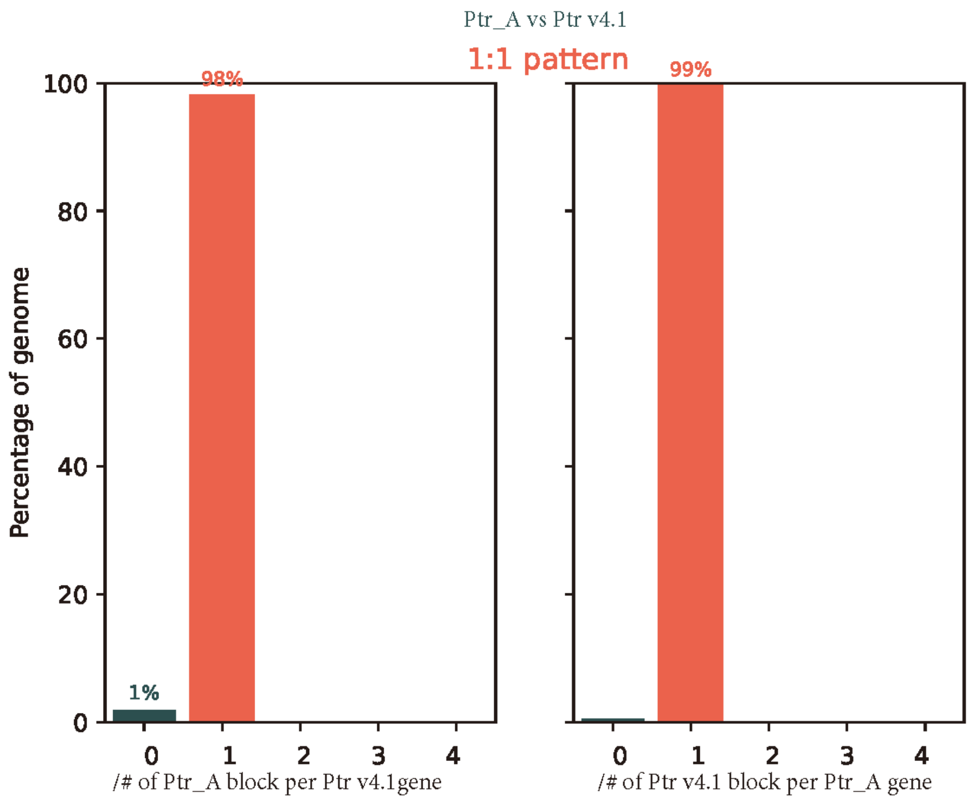

Supplemental Figure S13. Syntenic depth ratio analyses of Ptr_A and Ptr v4.1

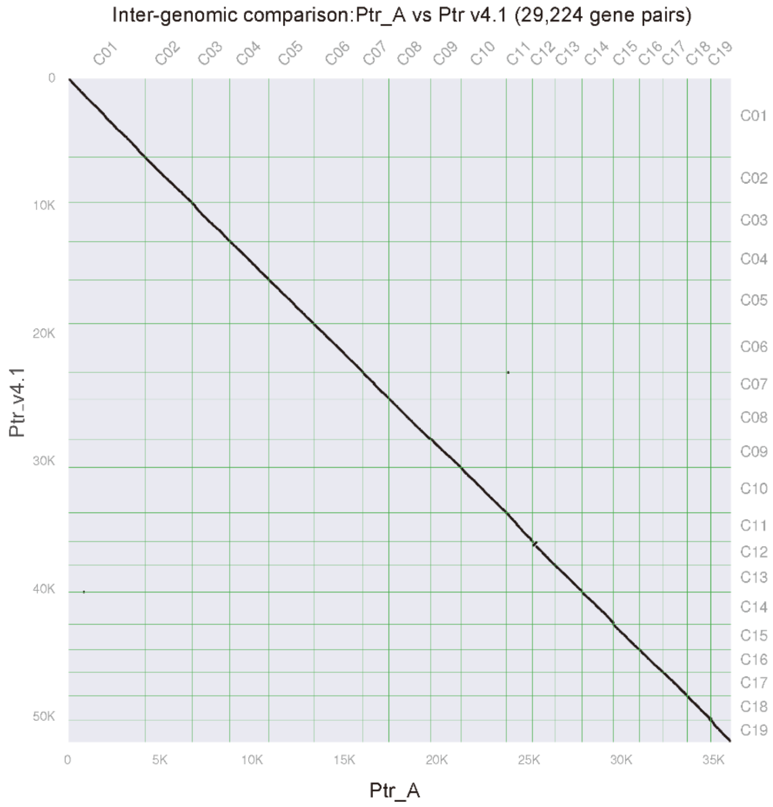

Supplemental Figure S14. Genome-wide syntenic relationship between Ptr_A and Ptr v4.1 assemblies (29,224 gene pairs)

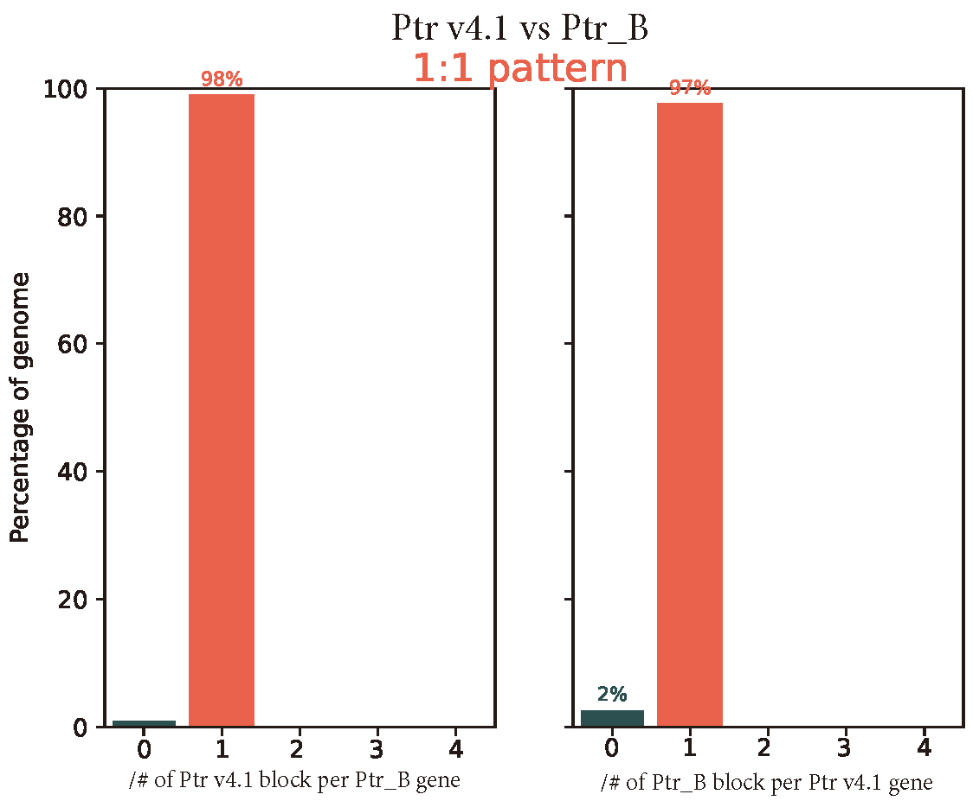

Supplemental Figure S15. Syntenic depth ratio analyses of Ptr v4.1 and Ptr_B

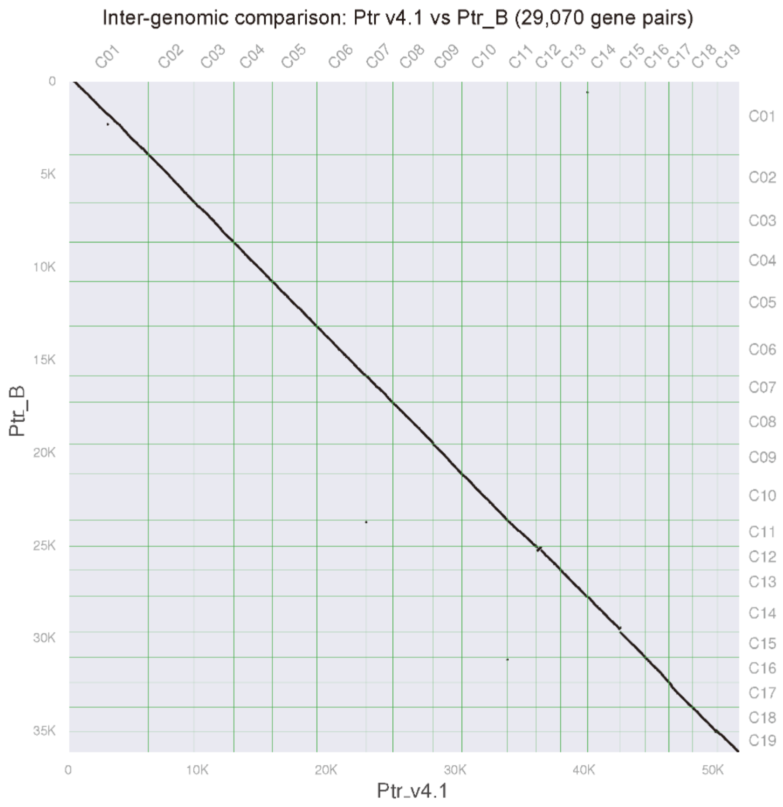

Supplemental Figure S16. Genome-wide syntenic relationship between Ptr_A and Ptr v4.1 assemblies (29,070 gene pairs)

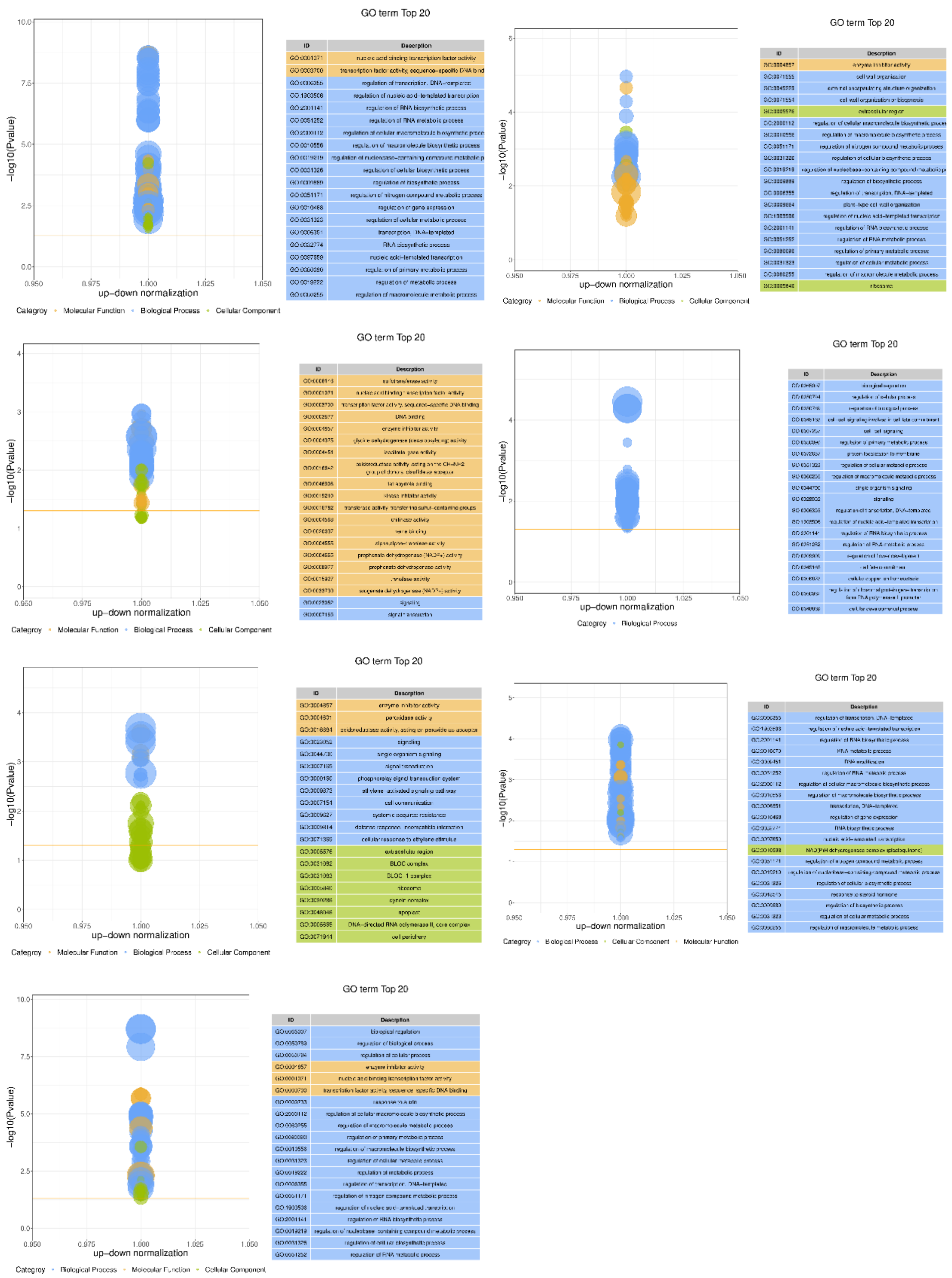

Supplemental Figure S17. A Go term top 20 in issues (Fiber, Leaf, Phloem, Root, Shoot, Vessel, Xylem)

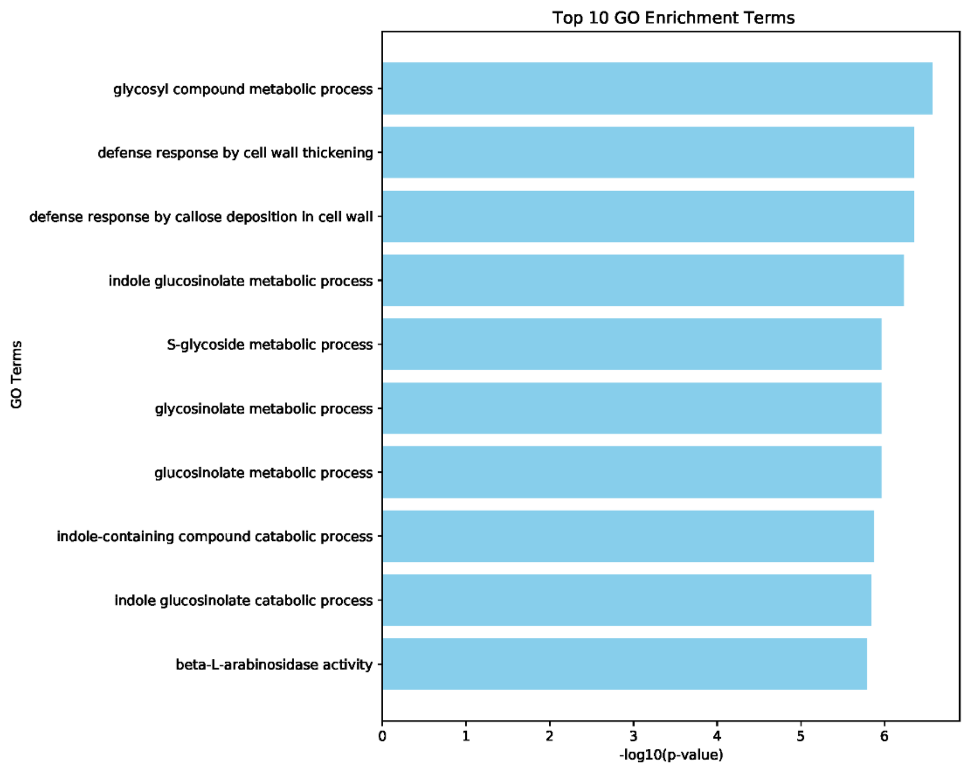

Supplemental Figure S18. Pathway Enrichment Analysis of Haplotype-Specific Alleles in *Populous trichocarpa* Using GO Database

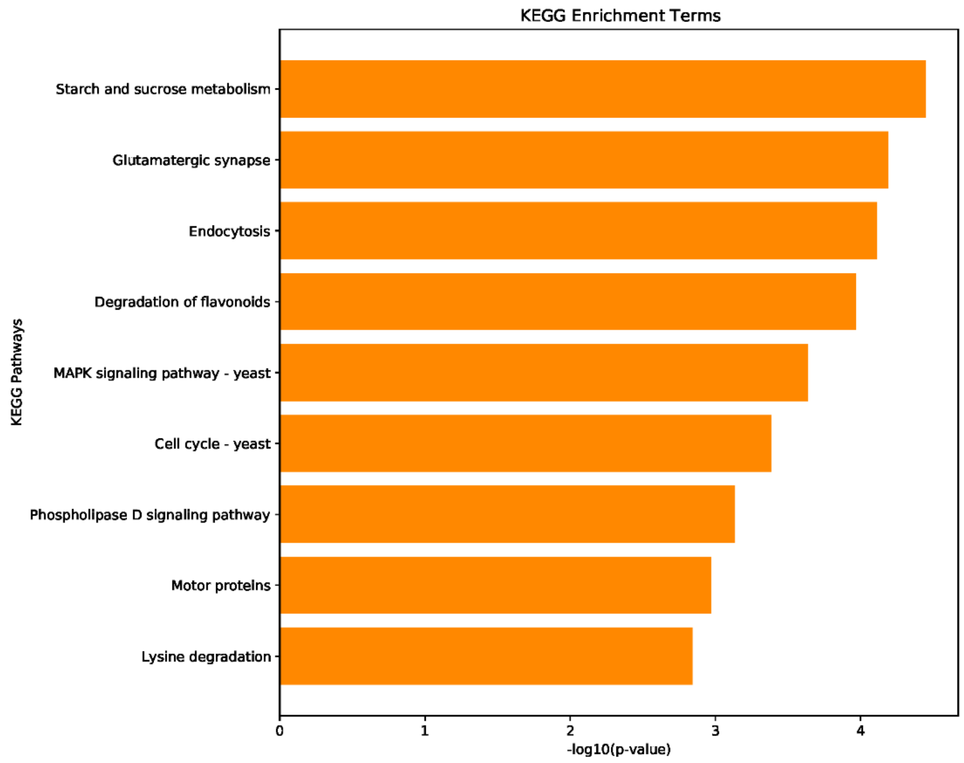

Supplemental Figure S19. Pathway Enrichment Analysis of Haplotype-Specific Alleles in *Populous trichocarpa* Using KEGG Database

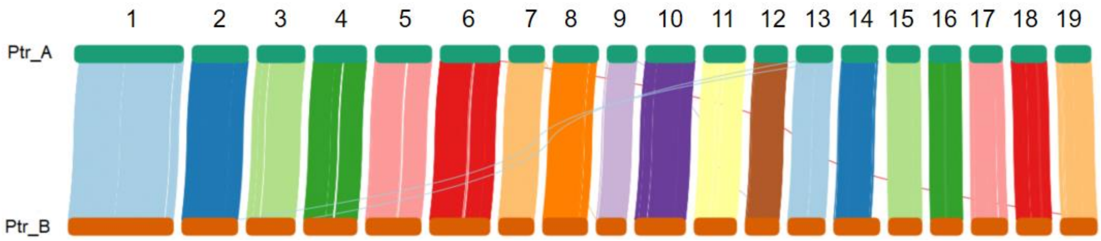

Supplemental Figure S20. Chromosomal Synteny Analysis between Ptr_A and Ptr_B Genomes

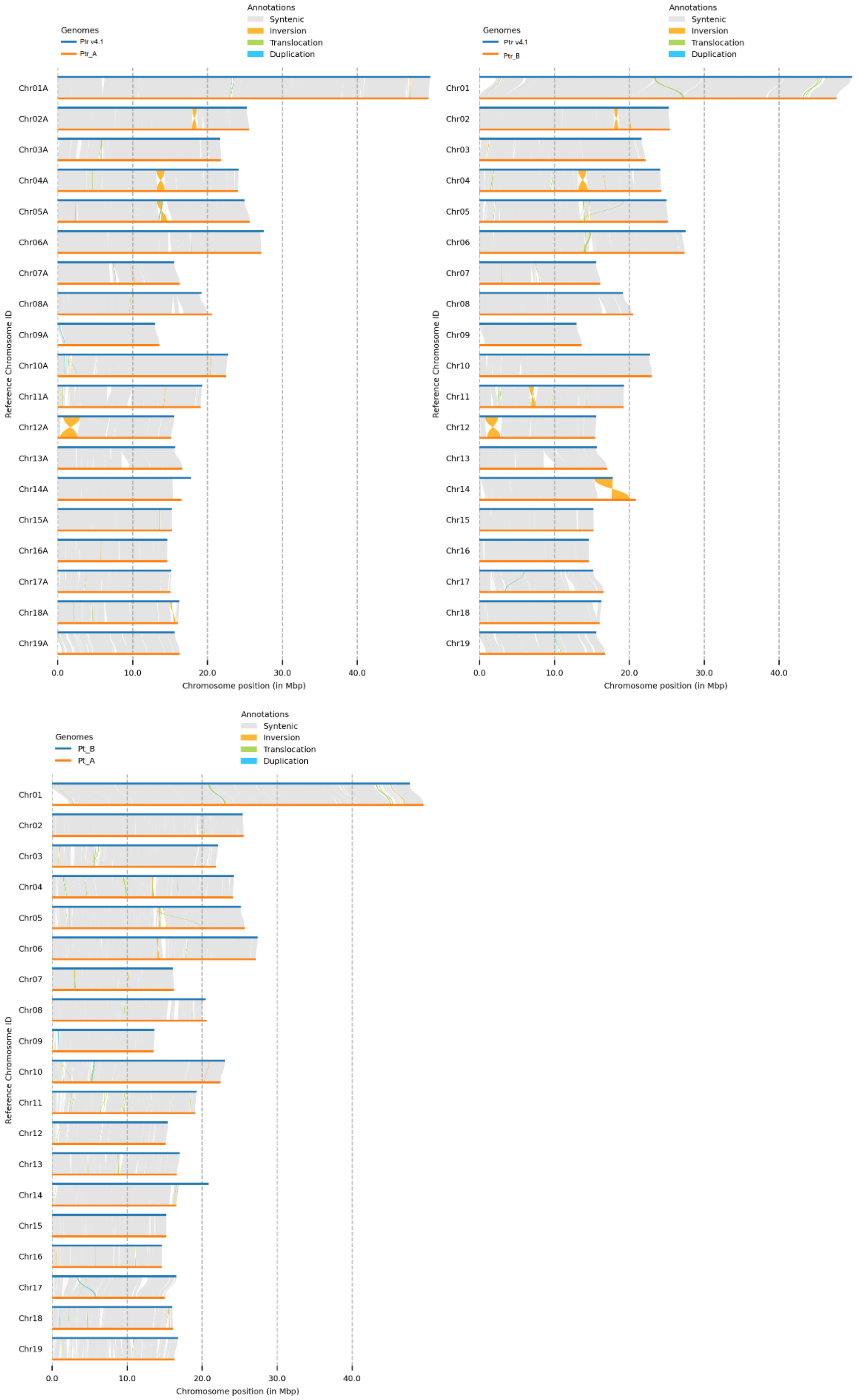

Supplemental Figure S21. Genome collinearity analysis between Ptr v4.1 , Ptr_A and Ptr_B haplotype genomes

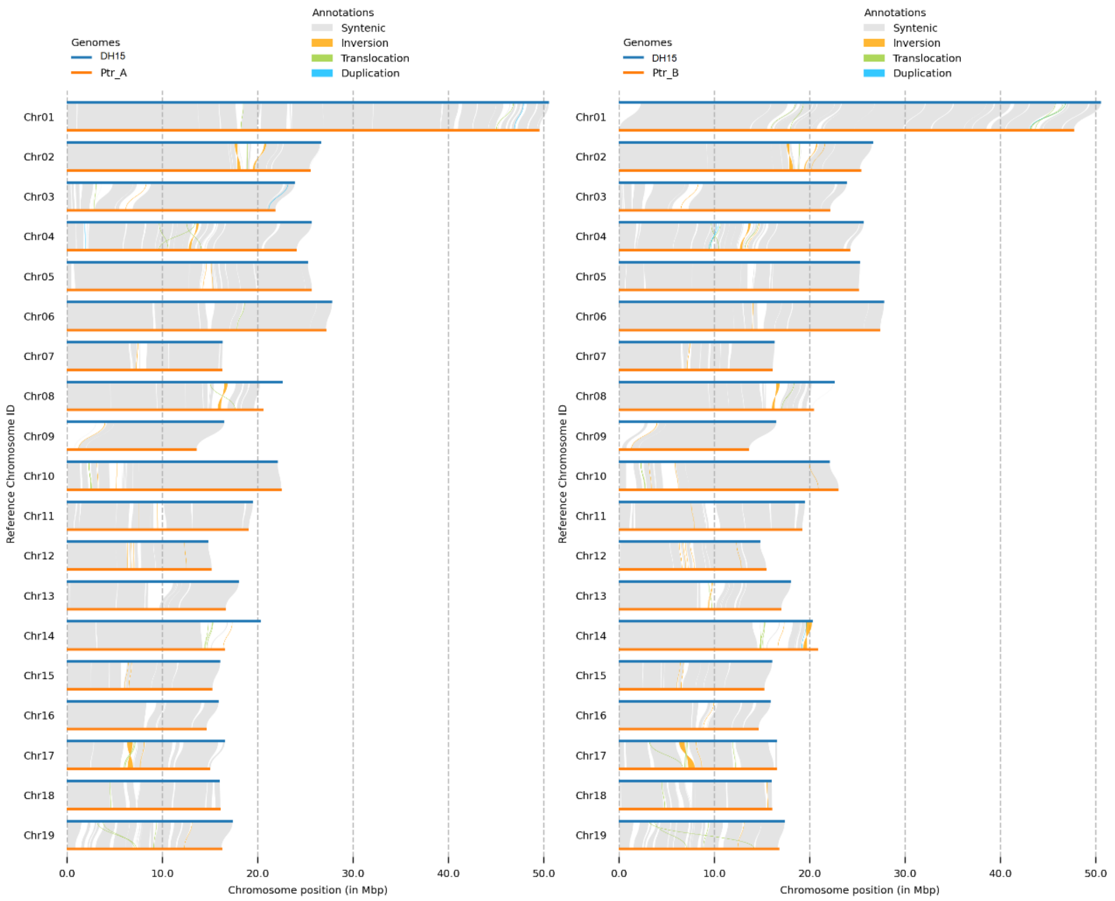

Supplemental Figure S22. Genome collinearity analysis between a doubled haploid line of *Populus ussuriensis* (DH15) and Ptr_A/Ptr_B haplotype genomes

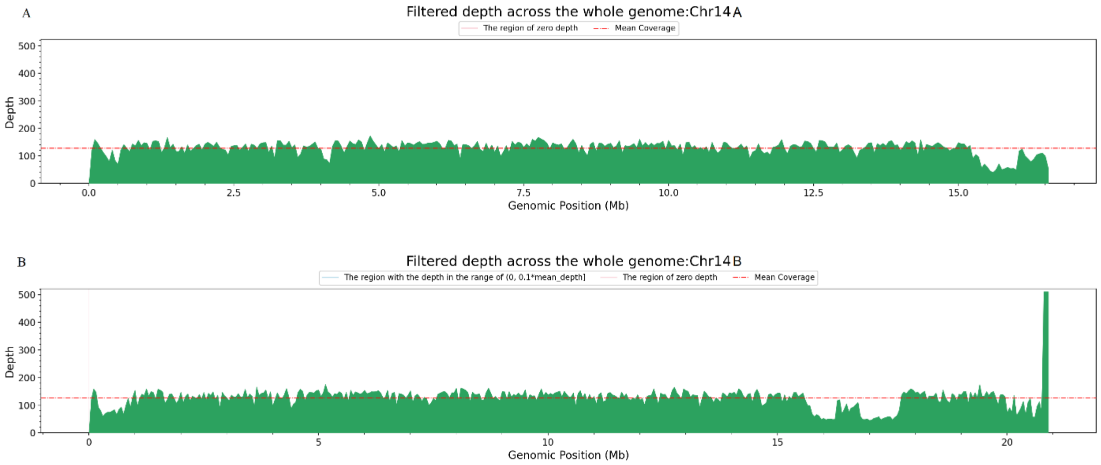

Supplemental Figure S23. GCI (Genome Continuity Inspector) evaluation results for Chr14A and Chr14B assemblies

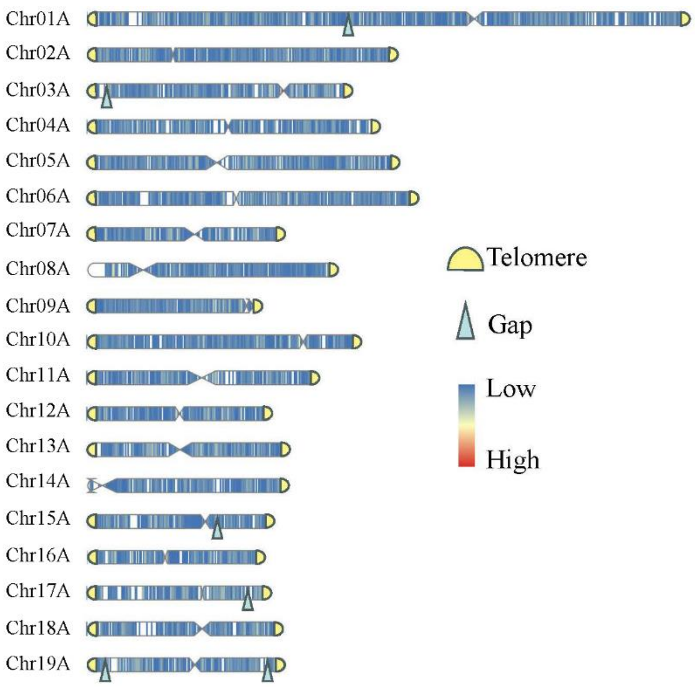

Supplemental Figure S24. SNP density of Ptr_A relative to Ptr v4.1 reference genome. The SNP density plot was generated based on the synteny analysis between Ptr_A and Ptr v4.1 genomes using SyRI, with Ptr v4.1 as the reference genome, followed by functional annotation of identified SNPs using SnpEff.

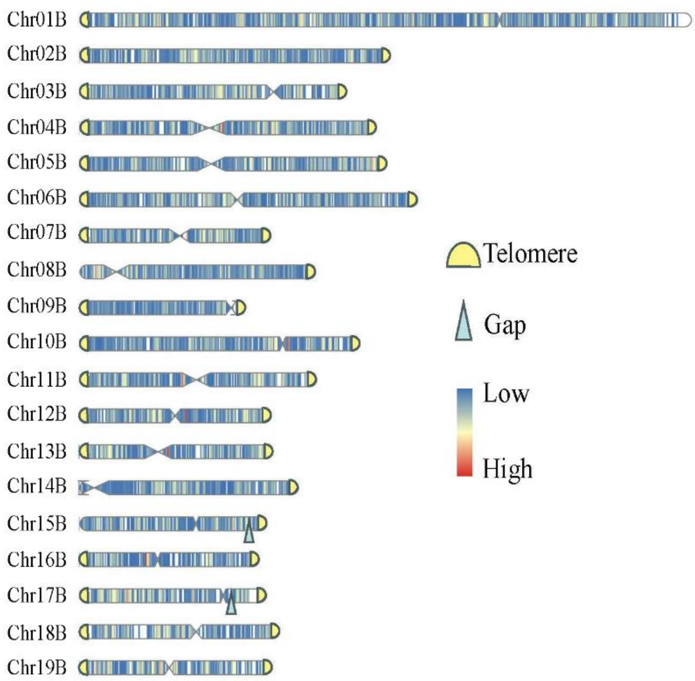

Supplemental Figure S25. SNP density of Ptr_B relative to Ptr v4.1 reference genome. The SNP density plot was generated based on the synteny analysis between Ptr_B and Ptr v4.1 genomes using SyRI, with Ptr v4.1 as the reference genome, followed by functional annotation of identified SNPs using SnpEff.

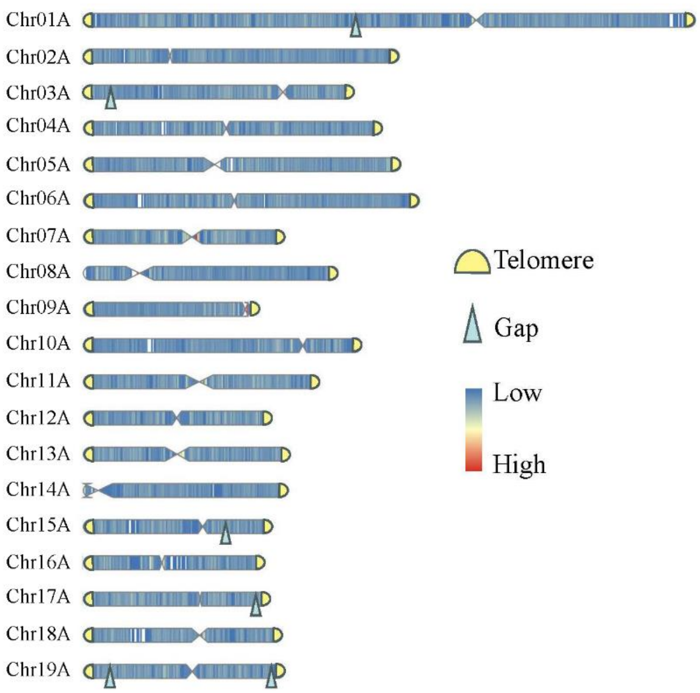

Supplemental Figure S26. SNP density of Ptr_A relative to Ptr_B reference genome. The SNP density plot was generated based on the synteny analysis between Ptr_A and Ptr_B genomes using SyRI, with Ptr_B as the reference genome, followed by functional annotation of identified SNPs using SnpEff.

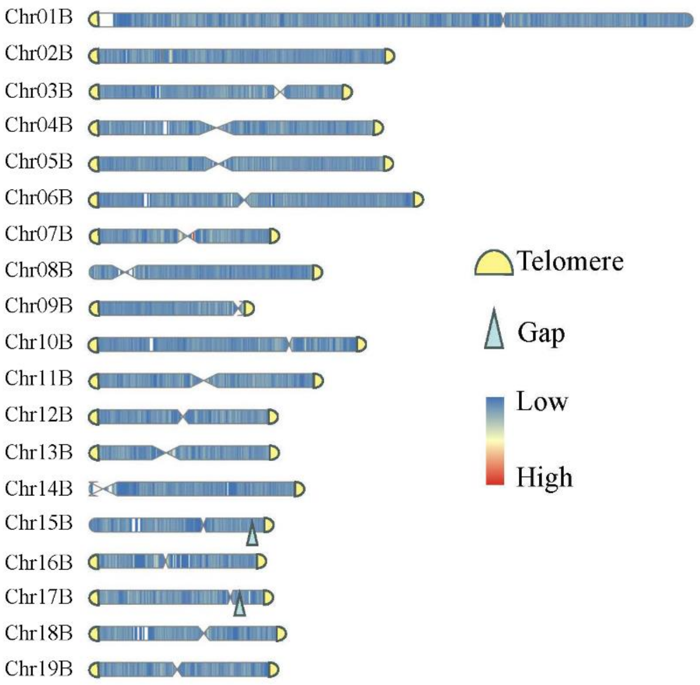

Supplemental Figure S27. SNP density of Ptr_B relative to Ptr_A reference genome. The SNP density plot was generated based on the synteny analysis between Ptr_A and Ptr_B genomes using SyRI, with Ptr_A as the reference genome, followed by functional annotation of identified SNPs using SnpEff.

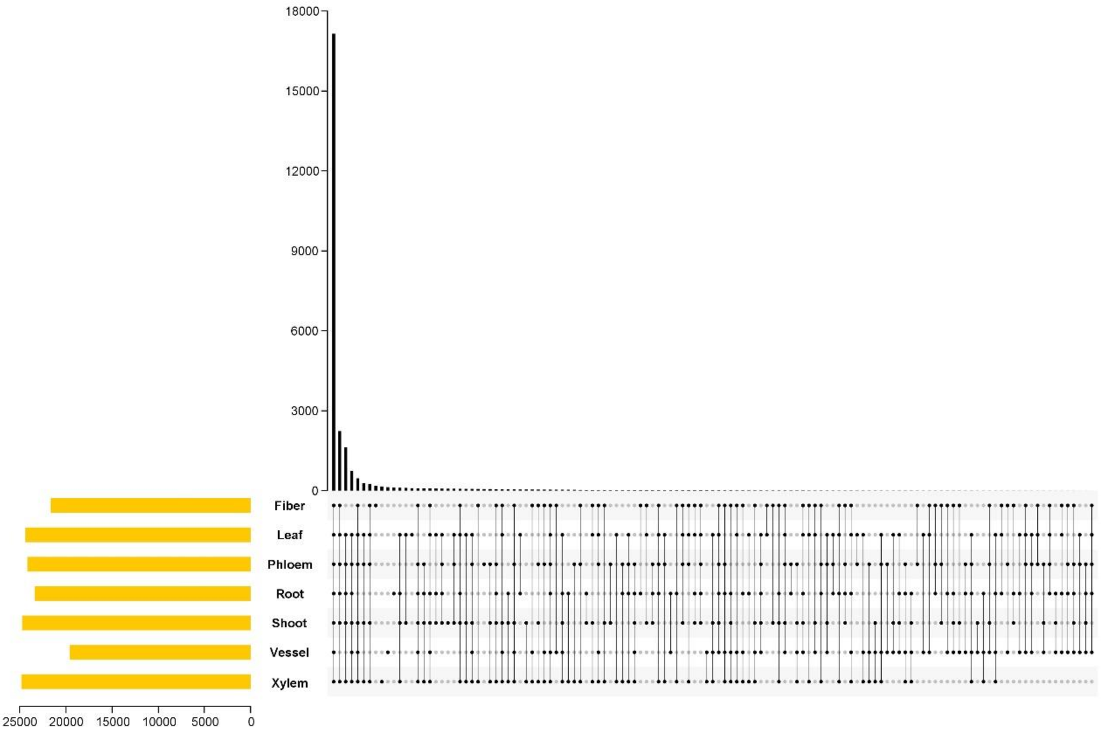

Supplemental Figure S28. Comparative analysis of gene expression patterns in different tissues

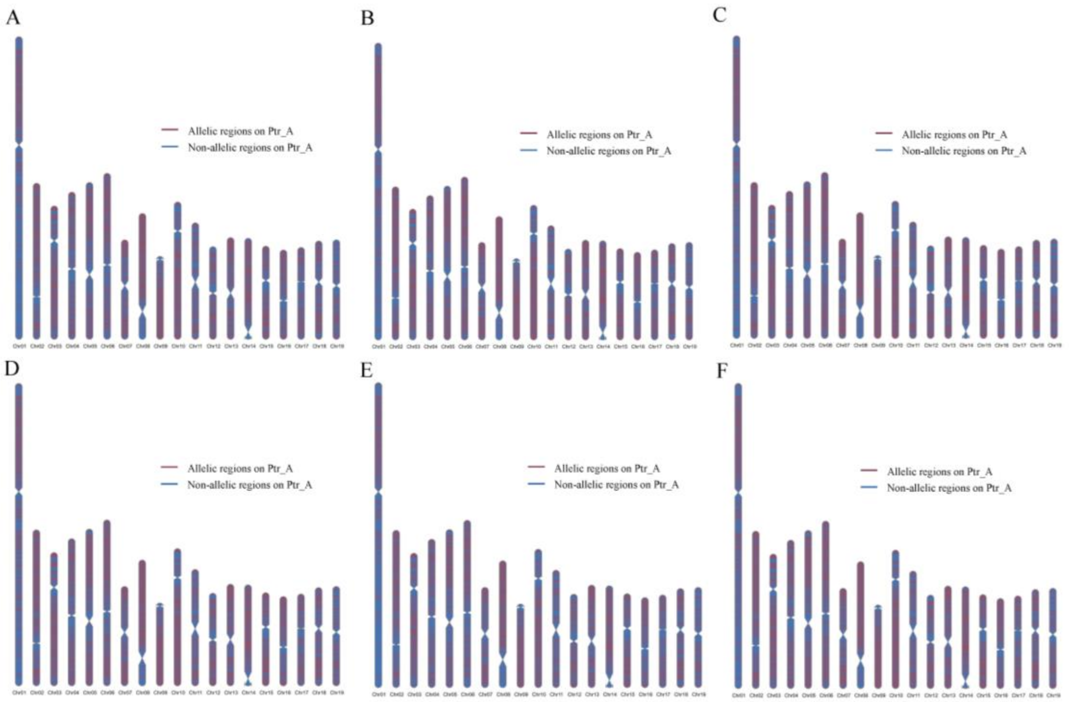

Supplemental Figure S29. Distribution of allelic regions across multiple tissues in Ptr_A. (A) Fiber tissue. (B) Leaf tissue. (C) Phloem tissue. (D) Shoot tissue. (E) Vessel tissue. (F) Xylem tissue.

Supplemental Tables

Supplemental Table S1. Statistics of HiFi sequencing data

Parameter	Value
Number of reads	3,634,344
Total bases (Gb)	59.98
Minimum length (bp)	86
Maximum length (bp)	49,075
Mean length (bp)	16,503
N50	16,502
GC content (%)	34

Supplemental Table S2. Statistics of Hi-C sequencing data

Parameter	R1	R2
Number of reads	836,010,492	836,010,492
Total bases (Gbp)	250.8	250.8
Read length (bp)	150	150
Q20 (%)	98.33	97.10
Q30 (%)	94.64	90.93
GC content (%)	35.40	35.32

Supplemental Table S3. Centromeres in Ptr_B genome

Chromosome	Start	End	Length (bp)
Chr01B	15,407,541	15,830,803	423,263
Chr02B	19,099,694	19,203,056	103,363
Chr03B	5,311,882	6,503,136	1,191,255
Chr04B	12,165,795	15,068,376	2,902,582
Chr05B	13,160,366	15,421,676	2,261,311
Chr06B	14,163,218	15,265,772	1,102,555
Chr07B	6,557,545	8,315,057	1,757,513
Chr08B	15,165,380	17,295,789	2,130,410
Chr09B	232,469	1,119,424	886,956
Chr10B	6,041,807	6,535,972	494,166
Chr11B	8,670,907	10,960,089	2,289,183
Chr12B	7,376,404	8,292,558	916,155
Chr13B	8,202,235	10,485,672	2,283,438
Chr14B	15,447,477	17,848,960	2,401,484
Chr15B	5,562,270	6,016,665	454,396
Chr16B	8,072,064	8,516,055	443,992
Chr17B	3,301,859	3,743,687	441,829
Chr18B	6,324,153	7,275,520	951,368
Chr19B	7,955,400	8,745,441	790,042

Supplemental Table S4. Busco of Ptr_A genome

Category	Count	Percent (%)
Complete BUSCOs (C)	1598	98.5
Complete and single-copy BUSCOs (S)	1333	82.6
Complete and duplicated BUSCOs (D)	256	15.9
Fragmented BUSCOs (F)	8	0.5
Missing BUSCOs (M)	17	1.0
Total BUSCO groups searched	1614	100

Supplemental Table S5. Busco of Ptr_B genome

Parameter	Count	Percent (%)
Complete BUSCOs (C)	1585	98.2
Complete and single-copy BUSCOs (S)	1323	82.0
Complete and duplicated BUSCOs (D)	262	16.2
Fragmented BUSCOs (F)	11	0.7
Missing BUSCOs (M)	18	1.1
Total BUSCO groups searched	1614	100

Supplemental Table S6. Overall Statistics of homologous gene identification and gene family analysis

Parameter	Value
Number of species	13
Number of genes	519,965
Number of genes in orthogroups	495,864
Number of unassigned genes	24,101
Percentage of genes in orthogroups (%)	95.4
Percentage of unassigned genes (%)	4.6
Number of orthogroups	31,086
Number of species-specific orthogroups	2,557
Number of genes in species-specific orthogroups	7,027
Percentage of genes in species-specific orthogroups (%)	1.4
Mean orthogroup size	16
Median orthogroup size	14
G50 (assigned genes)	25
G50 (all genes)	24
O50 (assigned genes)	6,843
O50 (all genes)	7,334
Number of orthogroups with all species present	13,119
Number of single-copy orthogroups	204

Supplemental Table S7. Comparative analysis between Ptr_A and the reference genome Ptr v4.1 by SyRI

Variation type	Count	Length of Ptr v4.1 (bp)	Length of Ptr_A (bp)
Syntenic regions	907	367,891,657	367,679,714
Inversions	41	5,152,930	4,920,533
Translocations	646	3,276,567	3,323,348
SNPs	1,586,306	1,586,306	1,586,306
Insertions	211904	3,033,565	-
Deletions	194,539	-	2,774,256

Supplemental Table S8. Comparative analysis between Ptr_B and the reference genome Ptr v4.1 by SyRI

Variation type	Count	Length of Ptr v4.1 (bp)	Length of Ptr_B (bp)
Syntenic regions	970	368,378,032	366,046,762
Inversions	42	6,495,586	6,739,959
Translocations	716	4,308,526	4,275,405
SNPs	1,631,616	1,631,616	1,631,616
Insertions	228,787	2,960,352	-
Deletions	204,659	-	2,873,022

Supplemental Table S9. Comparative analysis between Ptr_A and Ptr_B by SyRI

	Variation type	Count	Length of Reference (bp)	Length of Query (bp)
Ptr_A vs Ptr_B	Syntenic regions	2,612	361,420,827	3,61956,694
	Inversions	78	951,312	895,420
	Translocations	2,013	6,333,345	6,369,754
	SNPs	3,343,223	3,343,223	3,343,223
	Insertions	393,208	-	5,278,076
	Deletions	386,414	5,498,334	-
Ptr_B vs Ptr_A	Syntenic regions	2,612	361,840,867	361,214,116
	Inversions	74	901,163	951,412
	Translocations	2,011	6,408,474	6,360,653
	SNPs	3,339,802	3,339,802	3,339,802
	Insertions	394,717	-	5,226,768
	Deletions	385,128	5,342,554	-

Supplemental Table S10. Comparative analysis between Ptr_A and a doubled haploid line of *Populus ussuriensis* (DH15) by SyRI

Variation type	Count	Length of Ptr_A (bp)	Length of DH15 (bp)
Syntenic regions	4750	320,585,647	319,087,351
Inversions	113	5,256,400	5,191,884
Translocations	3,639	11,923,615	11,926,682
SNPs	7,665,592	7,665,592	7,665,592
Insertions	880,148	7,847,349	-
Deletions	843,966	-	7,378,304

Supplemental Table S11. Comparative analysis between Ptr_B and a doubled haploid line of *Populus ussuriensis* (DH15) by SyRI

Variation type	Count	Length of Ptr_B (bp)	Length of DH15 (bp)
Syntenic regions	4769	319,898,165	318,443,315
Inversions	119	6,365,326	5,955,075
Translocations	3,728	12,445,913	12,376,721
SNPs	7,674,708	7,674,708	7,674,708
Insertions	878,598	7,569,109	-
Deletions	841,870	-	7,366,247

Supplemental Table S12. SnpEff annotation summary of SNPs of Ptr_A and Ptr_B

	Type	Count	Percent (%)
Ptr_A	High	**500**	**0.015**
	Low	43,074	1.288
	Moderate	50,404	1.507
	Modifier	3,249,245	**97.127**
	Total	3,343,223	100
Ptr_B	High	**459**	**0.014**
	Low	42,944	1.285
	Moderate	50,676	1.517
	Modifier	**3,245,723**	**97.184**
	Total	3,339,802	100
